# An annotated checklist of mammals of Kenya

**DOI:** 10.24272/j.issn.2095-8137.2018.059

**Published:** 2018-10-17

**Authors:** Simon Musila, Ara Monadjem, Paul W. Webala, Bruce D. Patterson, Rainer Hutterer, Yvonne A. De Jong, Thomas M. Butynski, Geoffrey Mwangi, Zhong-Zheng Chen, Xue-Long Jiang

**Affiliations:** 1 Mammalogy Section, Department of Zoology, National Museums of Kenya, Nairobi 40658-00100, Kenya; 2 Department of Biological Sciences, University of Swaziland, Kwaluseni M201, Swaziland; 3 Mammal Research Institute, Department of Zoology & Entomology, University of Pretoria, Pretoria 0002, South Africa; 4 Department of Forestry and Wildlife Management, Maasai Mara University, Narok 861-20500, Kenya; 5 Integrative Research Center, Field Museum of Natural History, Chicago IL 60605-2496, USA; 6 Zoologisches Forschungsmuseum Alexander Koenig, Leibniz-Institut für Biodiversität der Tiere, Bonn 53113, Germany; 7 Eastern Africa Primate Diversity and Conservation Program, Nanyuki 149-10400, Kenya; 8 School of Natural Resources and Environmental Studies, Karatina University, Karatina 1957-10101, Kenya; 9 Sino-African Joint Research Center, Chinese Academy of Sciences, Nairobi 62000-00200, Kenya; 10 Kunming Institute of Zoology, Chinese Academy of Sciences, Kunming Yunnan 650223, China

**Keywords:** Checklist, Rodents, Bats, Ungulate, Carnivores, Shrews, Kenya

## Abstract

Kenya has a rich mammalian fauna. We reviewed recently published books and papers including the six volumes of *Mammals of Africa* to develop an up-to-date annotated checklist of all mammals recorded from Kenya. A total of 390 species have been identified in the country, including 106 species of rodents, 104 species of bats, 63 species of even-toed ungulates (including whales and dolphins), 36 species of insectivores and carnivores, 19 species of primates, five species of elephant shrews, four species of hyraxes and odd-toed ungulates, three species of afrosoricids, pangolins, and hares, and one species of aardvark, elephant, sirenian and hedgehog. The number of species in this checklist is expected to increase with additional surveys and as the taxonomic status of small mammals (e.g., bats, shrews and rodents) becomes better understood.

## INTRODUCTION

Kenya lies astride the equator on the eastern coast of Africa. It is a medium-sized country, covering an area of about 582 646 km^2^. The geography of Kenya is highly diverse, with various landforms that include coastline, lake basins, plains, hills, high mountains, and deserts. Similarly, the Kenyan climate is diverse and varies with geographical location. For example, rainfall and temperature are influenced by changes in altitude and distance to the coast and Lake Victoria. The Kenyan coast (0–100 m a.s.l.) is warm and humid, receiving about 1 000 mm of rainfall per year; the central highlands (1 000–2 500 m a.s.l.) are cool and humid, receiving the highest rainfall (over 2 000 mm per year) in Kenya; the hot and dry regions of northern and eastern Kenya (200–700 m a.s.l.) receive the lowest rainfall (<300 mm per year) (Bennun & Njoroge, 1999). Frost regularly occurs above 2 400 m a.s.l., with the hottest area (mean maximum temperature of 34 °C) being Lake Turkana (Bennun & Njoroge, 1999). The variations in rainfall, temperature, topography and landuse together influence the biomes and ecoregions of Kenya. Due to the complex topography, vegetation types and variation in climate, Kenya harbors a large diversity of organisms, with about 25 000 species of fauna and 7 000 species of flora currently recorded, along with at least 2 000 species of fungi and bacteria (NBU, 1992).

Over 10% of the country’s land area is presently gazetted as a national park, national reserve or forest reserve (Bennun & Njoroge, 1999). However, these protected areas were primarily established because they: 1) contain considerable populations of ‘big game’ (i.e., large mammals), which attract visitors; (2) are important water catchment areas; (3) support valuable timber for exploitation; or (4) contain few resident people at the time of establishment (Bennun & Njoroge, 1999). Thus, these protected areas were typically not established for the conservation of Kenya’s many smaller mammalian species such as hedgehogs, bats, rodents, otter-shrews, shrews, hares and elephant-shrews. More than 80% of Kenya’s land area is not under legal protection and is predominantly comprised of degraded vegetation, agriculture and settlement, resulting in ongoing loss of suitable natural habitats for mammals. However, a small but significant proportion of the unprotected zones are conserved as privately or community owned ranches and conservancies, which can support relatively undisturbed natural habitats, providing important refuges for some mammals. For many of Kenya’s protected areas, conservancies and ranches, only checklists of larger mammals are typically available. The remoteness, difficulty of access and security concerns in northern and some parts of eastern Kenya have made this region difficult to survey. For example, an apparently new giant sengi (*Rhynchocyon* sp.) was recorded in the Boni-Dodori Forest (Andanje et al., 2010) but the risk of attack and kidnappings by Somalia-based Al-Shabaab militants has prevented any further research on the species. The study of small mammals in Kenya, as elsewhere on the continent, is also hampered by a shortage of experienced taxonomists. Hence, compared with large mammals, there is a dearth of knowledge on the distribution and ecology of small mammals in Kenya.

Species checklists constitute alpha-diversity descriptors of the taxonomic richness of a given country (Whittaker, 1972), and are important tools for the effective conservation of threatened species at the national level (Amori et al., 2011). Past checklists of Kenyan mammals were published in the 1920s to early 1990s. These included checklists for Insectívora, Chiroptera, and Carnivora (Hollister, 1918), Chiroptera (Aggundey & Schlitter, 1984), Insectivora and Macroscelidea (Aggundey & Schlitter, 1986), Kingdon’s volumes of East African Mammals (Kingdon, 1974a, 1974b, 1977, 1982a, 1982b, 1984), Kingdon’s Field Guide to African Mammals (Kingdon, 1997), Mammals of East Africa, including Kenya (Davies & Vanden Berghe, 1994), and Key to Bats of East Africa (Patterson & Webala, 2012). However, these checklists are now outdated and incomplete. In the past 24 years since the last checklist (Davies & Vanden Berghe, 1994), no attempt has been made to compile and publish a revised checklist of mammals in Kenya, even though a good deal of research has been conducted during the same period. Therefore, an updated complete checklist of mammal species is both warranted and of great conservation importance. This current checklist attempts to update the list of all mammals recorded in Kenya, and thus reflects recent advances in research of Kenyan mammals.

## METHOD OF CHECKLIST PREPARATION

The current checklist was compiled by reviewing the six volumes of the *Mammals of Africa* (MOA): Vol. 1 (Introductory Chapters and Afrotheria: Kingdon et al., 2013), Vol. 2 (Primates: Butynski et al., 2013), Vol. 3 (Rodents, Hares and Rabbits: Happold D, 2013a), Vol. 4 (Hedgehogs, Shrews and Bats: Happold M & Happold D, 2013), Vol. 5 (Pigs, Hippopotamuses, Chevrotain, Giraffes, Deer and Bovids: Kingdon & Hoffman, 2013a), and Vol. 6 (Carnivores, Pangolins, Equids and Rhinoceroses: Kingdon & Hoffman, 2013b); *Rodents of Sub-Saharan Africa: A Biogeographic and Taxonomic Synthesis* (Monadjem et al., 2015), *Keys to the Bats (Mammalia: Chiroptera) of East Africa* (Patterson & Webala, 2012), and the IUCN website (IUCN Red List of Threatened Species, 2017) (only for Cetaceans). Prior changes in the taxonomy of each taxon are not included in this checklist because respective volumes of the MOA provide detailed information on the same. However, changes stemming from more recent literature are noted. Nomenclature follows the MOA, except where noted. We recognized the families Miniopteridae (Miller-Butterworth et al., 2007) and Rhinonycteridae (Foley et al., 2015) for bats and Heterocephalidae for rodents (Patterson & Upham, 2014). The checklist, which covers both terrestrial and aquatic (freshwater and marine) species, is presented by order, family, genus, scientific name, species authority, preferred common English, Swahili (English Coastal Swahili Dictionary online (ECSDO), 2016; Kingdon, 1974a, 1974b, 1977, 1982a, 1982b, 1984, 1997), and Chinese names (mostly following Wang et al., 2001), distributional range in Africa and Kenya, and the broad habitat types where it occurs. Cetacean species (IUCN Red List of Threatened Species, 2017) were included in the list if their distribution range encompassed the shallow marine habitat over the continental shelf and deep sea of the Indian Ocean along Kenya’s coastline. Introduced species without a wild breeding population, those not confirmed to occur in Kenya, and those locally extinct are not included in the list.

## CHECKLIST OF MAMMALS

**ORDER HYRACOIDEA (Hyraxes–four species)**

**Family PROCAVIIDAE**

**Genus** ***Dendrohyrax*** Gray, 1868. Tree Hyraxes

1. ***Dendrohyrax arboreus*** A. Smith, 1827. English: Southern Tree Hyrax. Swahili: Perere. Chinese: 南树蹄兔. Recorded from southern and eastern Africa, including E and SE DR Congo. Well-developed woodlands or forests. In Kenya, recorded from W-S Kenya along the Kenya-Tanzania border, as well as C Kenya (Milner & Gayland, 2013).
2. ***Dendrohyrax validus*** True, 1890. English: Eastern Tree Hyrax. Swahili: Perere. Chinese: 坦桑树蹄兔. Recorded only from Kenya and Tanzania. Moist lowland and montane forests, and in a wide altitudinal range from sea level to 3070 m a.s.l. on Mt Kilimanjaro. In Kenya, recorded from the SE (Taita Hills) (Roberts et al., 2013).

**Genus** ***Heterohyrax*** Gray, 1868. Bush Hyraxes

3. ***Heterohyrax brucei*** Gray, 1868. English: Bush Hyrax; Yellow-spotted Hyrax. Swahili: Perere Mawe; Pimbi Madoa. Chinese: 小齿蹄兔. Recorded from Sudan and Eritrea from the east and Horn of Africa to South Africa, as well as SW DR Congo. In rocky kopjes, sheer cliffs or precipices, and piles of large boulders with openings, as well as in forests along rivers. In Kenya, widespread (Barry & Hoeck, 2013).

**Genus** ***Procavia*** Storr, 1780. Rock Hyraxes

4. ***Procavia capensis*** (Pallas, 1766). English: Rock Hyrax. Swahili: Pimbi/Pimbe. Chinese: 岩蹄兔. Recorded from S Mauritania through S Algeria and Libya to Egypt, western through to East Africa, including the Horn of Africa, as well as southern Africa. In a wide range of habitats, from arid deserts to rainforest, and from sea level to the alpine zone of Mt Kenya (3 200–4 300 m a.s.l.). In Kenya, widely distributed (Hoeck & Bloomer, 2013).

**ORDER PROBOSCIDEA (African Elephant–one species)**

**Family ELEPHANTIDAE**

**Genus** ***Loxodonta*** Anonymous, 1827. African Elephant

5. ***Loxodonta africana*** Blumenbach, 1797. English: African Bush Elephant. Swahili: Ndovu; Tembo. Chinese: 非洲草原象. Recorded from many countries in Africa S of the Sahara. In a wide variety of habitats, including sub-deserts to swamps, lowland rainforests, gallery and montane forests, upland moors, flood-plains, savannas and various woodlands. Widespread in Kenya (Poole et al., 2013).

**ORDER SIRENIA (Dugongs–one species)**

**Family DUGONGIDAE**

**Genus** ***Dugong*** Lacépède, 1799. Dugongs

6. ***Dugong dugon*** Müller, 1776. English: Dugong. Swahili: Nguva. Chinese: 儒艮. Recorded from the Red Sea in Egypt through the Gulf of Aden to Mozambique. In wide shallow protected bays, wide shallow mangrove channels and large inshore islands over the continental shelf. In Kenya, recorded along the coast from the border with Tanzania to Somalia (Marsh & Dutton, 2013).

**ORDER AFROSORICIDA (Tenrecs and Golden Moles–three species)**

**Family TENRECIDAE**

**Genus** ***Potamogale*** Du Chaillu, 1860. Giant Otter-shrew

7. ***Potamogale velox*** du Chaillu, 1860. English: Giant Otter-shrew; Swahili: unavailable. Chinese: 大獭鼩. Recorded from E Nigeria and Cameroon, Gabon, DR Congo, N Angola, W Uganda and extreme W-C Kenya. Small, slow flowing streams in equatorial rainforests, forest pools and mountain torrents. In Kenya, recorded only in Kakamega (Mt. Elgon, Cherangani Hills) (Vogel, 2013).

**Family CHRYSOCHLORIDAE**

**Genus** ***Chrysochloris*** Lacépède, 1799. Golden-moles

8. ***Chrysochloris stuhlmanni*** Matschie, 1894. English: Stuhlmann’s Golden-mole; Swahili: unavailable. Chinese: 斯氏金毛鼹. Recorded in small fragmented populations in Cameroon, E DR Congo, Uganda, Kenya and Tanzania. Montane habitats, including grasslands, bamboo thickets, ericaceous vegetation, and *Podocarpus* and *Hagenia/Hypericum* woodlands (Bronner, 2013). In Kenya, recorded only on Mt. Elgon and the Cherangani Hills (Bronner, 2013).
9. ***Chrysochloris fosteri*** (St. Leger, 1931). English: Elgon Golden-mole; Swahili: unavailable. Chinese: 埃尔贡金毛鼹. Previously included in *Chrysochloris stuhlmanni* Matschie, but distinctly larger than any other species in that genus. Thorn and Kerbis Peterhans (2009) elevated it to species level. Montane habitats in Kenya and Uganda up to 4 000 m a.s.l. In Kenya, recorded only on Mt. Elgon and the Cherangani Hills (Bronner, 2013 as *Chrysochloris stuhlmanni*).

**ORDER MACROSCELIDEA (Sengis–five species)**

**Family MACROSCELIDIDAE**

**Genus** ***Elephantulus*** Thomas and Schwann, 1906. Sengis

10. ***Elephantulus brachyrhynchus*** A. Smith, 1836. English: Short-snouted Sengi. Swahili: Sengi. Chinese: 短吻象鼩. Recorded throughout eastern and southern Africa, from Uganda and Kenya to S DR Congo, Angola, Zambia, Malawi, Zimbabwe and N South Africa. Savanna habitats with thick cover. In Kenya, widespread mostly W of the Rift Valley (Perrin, 2013a).
11. ***Elephantulus rufescens*** Peters 1878. English: Rufous Sengi. Swahili: Sengi. Chinese: 赤褐象鼩. Recorded from SE South Sudan, E Ethiopia, Somalia, E Uganda, Kenya and Tanzania. Dry woodlands and bushlands, open wooded steppe and grasslands. In Kenya, widespread in drier habitats (Perrin & Rathbun, 2013).

**Genus** ***Petrodromus*** Peters, 1846. Four-toed Sengis

12. ***Petrodromus tetradactylus*** Peters, 1846. English: Four-toed Sengi. Swahili: Isanje. Chinese: 四趾岩象鼩. Recorded in DR Congo (S of Zaire River) and in Kenya, Tanzania, Zambia, Malawi, Mozambique, SE Zimbabwe and N South Africa. Woody thickets in forests, woodlands and rocky habitats. In Kenya, recorded only in the SE (Rathbun, 2013a).

**Genus** ***Rhynchocyon*** Peters, 1847. Giant Sengis

13. ***Rhynchocyon chrysopygus*** Günther, 1881. English: Golden-rumped Giant Sengi. Swahili: Njule ya Gedi/Fugu. Chinese: 黄臀象鼩. Endemic to Kenya. Coastal semi-decidous forests, woodlands with thick canopy and coastal rag scrub. In Kenya, recorded in small area S of the Tana River to the Arabuko-Sokoke Forest and Rabai near Mombasa (Rathbun, 2013b).
14. ***Rhynchocyon petersi*** Bocage, 1880. English: Black-and-Rufous Giant Sengi. Swahili Sengi/Njule. Chinese: 黑象鼩. Recorded in only a few localities in coastal S Kenya and NE Tanzania (also Zanzibar and Mafia Islands). Evergreen semi-deciduous forests, dense woodlands, coral rag scrub and overgrown agricultural lands. In Kenya, recorded in several forests S of Mombasa (Shimba Hills, Mrima, Marenji, Gongoni and Dzombo Forests) (Rathbun, 2013c).

**ORDER TUBULIDENTATA (Aardvark–one species)**

**Family ORYCTEROPODIDAE**

**Genus** ***Orycteropus*** G. Cuvier, 1798. Aardvark

15. ***Orycteropus afer*** Pallas, 1766. English: Aardvark. Swahili: Mhanga; Kukukifuku; Fundi-Mchanga. Chinese: 土豚. Recorded from many countries in Africa S of the Sahara. In a wide range of habitats, including semi-arid Karoo areas of Southern Africa, grasslands, all savanna types, rainforests, woodlands and thickets. In Kenya, widely distributed in dry and moist habitats with well-drained soils (Taylor, 2013).

**ORDER PRIMATES (Primates–19 species)**

**Family HOMINIDAE**

**Genus** ***Homo*** (Linnaeus, 1758). Humans

16. ***Homo sapiens*** Linnaeus, 1758. English: Modern Human. Swahili: Mtu. Chinese: 人. Most of the world, including all of Kenya (Kingdon, 2013).

**Family CERCOPITHECIDAE**

**Genus** ***Colobus*** Illiger, 1811. Black-and-White Colobus Monkeys

17. ***Colobus angolensis*** Sclater, 1860. English: Angola Colobus. Swahili: Mbega. Chinese: 安哥拉疣猴. Recorded from C Congo Basin, E to the Rwenzori Mts and L. Victoria, S-W Rwanda, W Burundi, and NW of Lake Tanganyika, as well as S-NW Angola. In montane, mid-altitude, lowland and coastal forests. One subspecies recognized in Kenya: *Colobus a. palliatus* Peters, 1868 Peters’s Angola Colobus. In Kenya, only recorded S of Mombasa (Shimba, Kinondo, Gongoni, Mrima, Nzombo and Marenji Forest) and other forests in the SE (Bocian & Anderson, 2013).
18. ***Colobus guereza*** Rüppel, 1835. English: Guereza Colobus. Swahili: Mbega. Chinese: 东黑白疣猴. Recorded from E Nigeria, N of the Congo Basin to E Africa, Gabon, Congo and E Ethiopia. In a wide array of forest types, including lowland and medium-altitude moist forest, montane forest, swamp forest, dry forest, gallery forest and disturbed forest. Four subspecies recognized in Kenya: *Colobus g. matschiei* Neumann, 1899, Mau Forest Guereza, recorded from CW Kenya, W of the Eastern Rift Valley; *Colobus g. kikuyuensis* Lönnberg, 1912, Mount Kenya Guereza, endemic to the Central Highlands of Kenya, E of the Eastern Rift Valley; *Colobus g. percivali* Heller, 1913, Mount Uarges Guereza, endemic to Mathews Range, C Kenya (Fashing & Oates, 2013); and *Colobus g. caudatus* Oldfield Thomas, 1885, Mount Kilimanjaro guereza, restricted to Kitobo and Loitokitok Forest Reserves (Butynski & De Jong, 2015).

**Genus** ***Procolobus*** de Rochebrune, 1887. Olive Colobus Monkey, Red Colobus Monkey

19. ***Procolobus rufomitratus*** (Peters, 1879). English: Eastern Red Colobus. Swahili: Mbega. Chinese: 东绿疣猴. Recorded from western, S-N Central African Republic, E Kenya, Southern Sudan, S-N Zambia and SW Tanzania. In forest-miombo mosaics, swamp, gallery, lowland and mid-altitude forests, montane moist forests and degraded secondary forests. One subspecies recognized in Kenya: *Procolobus r. rufomitratus* (Peters, 1879), Tana River Red Colobus. Recorded from SE Kenya in floodplain forests of the lower Tana River (Struhsaker & Grubb, 2013).

**Genus** ***Cercocebus*** É. Geoffroy, 1812. Drill-Mangabeys

20. ***Cercocebus galeritus*** Peters, 1879. English: Tana River Mangabey. Swahili: unavailable. Chinese: 塔纳白眉猴. Endemic to Kenya. In floodplain forests and adjacent woodland and bushland along the lower Tana River. In Kenya, recorded only from Kanjonja in the N to Tana Delta in the S (Wieczkowski & Butynski, 2013).

**Genus** ***Papio*** Erxleben, 1777. Baboons

21. ***Papio cynocephalus*** (Linnaeus, 1766). English: Yellow Baboon. Swahili: Nyani Njano. Chinese: 黄狒狒. Recorded from Angola, through S DR Congo, to E Kenya, SE Ethiopia, C Somalia, Tanzania, Malawi, Zambia and N Mozambique. In open miombo and savanna woodland. One subspecies recognized in Kenya: *Papio c. ibeanus* Thomas, 1893, Ibean Yellow Baboon. Recorded from SE Kenya (Altmann et al., 2013).
22. ***Papio anubis*** (Lesson, 1827). English: Olive Baboon. Swahili: Nyani. Chinese: 东非狒狒. Recorded from Mauritania to N Cameroon, E-C Ethiopia and SW lowlands of Eritrea, East Africa as well as SE DR Congo. In a wide variety of habitats but typically in open habitats. Widespread in W, C, N and SW Kenya (Palombit, 2013).

**Genus** ***Erythrocebus*** Trouessart, 1897. Patas Monkey

23. ***Erythrocebus patas*** (Schreber, 1775). English: Patas Monkey. Swahili: Kima. Chinese: 赤猴. Recorded from NW Senegal through Sudan to W Ethiopia to N DR Congo, and East Africa. In wooded savanna and woodland-grassland margins (Isbell, 2013). One subspecies recognized in Kenya: *Erythrocebus p. pyrrhonotus* (Hemprich & Ehrenberg, 1829), Eastern Patas Monkey. Patchily distributed in W, C and S Kenya (De Jong et al., 2008).

**Genus** ***Chlorocebus*** Gray, 1870. Savanna Monkeys

24. ***Chlorocebus tantalus*** (Ogilby, 1841). English: Tantalus Monkey. Swahili: Tumbili; Ngendere. Chinese: 坦塔罗斯绿猴. Recorded from Mali, Burkina Faso, Ghana, southern Sudan, NE DR Congo, N Uganda and NW Kenya. In a variety of habitats, including savanna woodlands, swamp forests, gallery forests and forest edge. One subspecies recognized in Kenya: *Chlorocebus t. budgetti* (Pocock, 1907), Budgett’s Tantalus. Recorded in SW of Kenya (W of Lake Turkana) (Nakagawa, 2013).
25. ***Chlorocebus pygerythrus*** (F. Cuvier, 1821). English: Vervet Monkey. Swahili: Tumbili; Ngendere. Chinese: 青腹绿猴. Recorded from S Somalia, S Ethiopia, east Africa, Malawi, Zambia, Mozambique, N and E Botswana and South Africa. In savanna-woodlands, primarily along water-courses, swamps and lakeshores. Two subspecies recognized in Kenya; *Chlorocebus p. excubutor* (Schwarz, 1926), Manda Vervet Monkey, recorded only in SE Kenya (Manda and Patta islands); and *Chlorocebus p. hilgerti* (Neumann, 1902), Hilgert’s Vervet Monkey, patchily distributed throughout most of Kenya (Isbell & Enstam-Jaffe, 2013).

**Genus** ***Cercopithecus*** Linnaeus, 1758. Arboreal Guenons

26. ***Cercopithecus neglectus*** Schlegel, 1876. English: De Brazza’s Monkey. Swahili: Kalasinga. Chinese: 德氏长尾猴. Recorded from E Cameroon, Equatorial Guinea, N Central African Republic, N Gabon, NE Angola, southern Sudan, SW Ethiopia and C Kenya. In riverine, gallery and swamp forests, including secondary forest. In Kenya, patchily distributed in SW and C regions (Mathews Range) (Gautier-Hion, 2013).
27. ***Cercopithecus mitis*** Wolf, 1822. English: Gentle Monkey. Swahili: Kima. Chinese: 青长尾猴. Recorded from W Angola, N Ethiopia, SE Sudan, S Somalia, East Africa, E DR Congo, NE Zambia, N Mozambique, Zimbabwe and South Africa. In a wide range of habitats, including lowland, mid-altitude, montane, riverine, gallery, coastland bamboo forests, bushland and woodland (Lawes et al., 2013). Four subspecies recognized in Kenya: *Cercopithecus m. albogularis* (Sykes, 1831), Zanzibar Sykes’s Monkey, recorded from SE Kenya, S of Galana River, W to Kibwezi and Tsavo West National Park (NP) (De Jong & Butynski, 2012); *Cercopithecus m. stuhlmanni* Matschie, 1893, Stuhlmann’s Blue Monkey, recorded from SW Kenya, W of the Eastern Rift Valley; *Cercopithecus m. albotorquatus* Pousargues, 1896, Pousargues’s Monkey, near-endemic to the N coast of Kenya, N to at least Boni National Reserve (NR) and Dodori NR, and inland along Tana River to Meru NP (De Jong & Butynski, 2011); and *Cercopithecus m. kolbi* Neumann, 1902, Kolb’s Monkey, endemic to the Kenyan Highlands, E of the Eastern Rift Valley.
28. ***Cercopithecus ascanius*** (Audebert, 1799). English: Red-tailed Monkey. Swahili: Kima. Chinese: 肯尼亚长尾猴. Recorded from N Angola, DR Congo and Central African Republic eastwards to W Kenya and NW Tanzania. In lowland, mid-elevation, montane, swamp, riverine and gallery forests, including secondary forests. One subspecies recognized in Kenya: *Cercopithecus a. schmidti* Matschie, 1892, Schmidt’s Red-tailed Monkey, recorded from SW Kenya, W of the Eastern Rift Valley (Cords & Sarmiento, 2013).

**Family LORISIDAE**

**Genus** ***Perodicticus*** Bennett, 1831. Pottos

29. ***Perodicticus potto*** (Müller, 1766). English: Potto. Swahili: Kami. Chinese: 树熊猴. Recorded from Upper Guinea, S in DR Congo to E and C Kenya. In lowland, mid-elevation, montane and swamp forests, including secondary forests (Pimley & Bearder, 2013). Two subspecies recognized in Kenya: *Perodicticus p. ibeanus* Thomas, 1910, Eastern Potto, recorded from SW Kenya (Butynski & De Jong, 2007); and *Perodicticus p. stockleyi* (Butynski & De Jong, 2007), Mount Kenya Potto, endemic to Mt. Kenya (Butynski & De Jong, 2007).

**Family GALAGIDAE**

**Genus** ***Otolemur*** Coquerel, 1859. Greater Galagos

30. ***Otolemur crassicaudatus*** (É. Geoffroy, 1812). English: Large-eared Greater Galago. Swahili: Komba. Chinese: 粗尾婴猴. Recorded from Angola, DR Congo, NW Tanzania, S Kenya, Malawi, Zambia, E Zimbabwe, E Botswana, E South Africa and Swaziland. In woodlands, savannas, thickets and forests (Bearder & Svoboda, 2013). Two subspecies recognized in Kenya: *Otolemur c. monteiri* (Bartlett in Gray, 1863), Miombo Silver Galago recorded from SE Kenya; and *Otolemur c. argentatus* (Lönnberg, 1913), Northern Silver Galago from SW Kenya.
31. ***Otolemur garnettii*** (Ogilby, 1838). English: Small-eared Greater Galago. Swahili: Komba Mkubwa. Chinese: 小耳大婴猴. Recorded from Somalia, C-SE Kenya, SE Tanzania and S-N Mozambique. In coastal, mid-elevation and montane forests (0–2 400 m a.s.l) and forest-agriculture mosaics (Harcourt & Perkin, 2013a). Three subspecies recognized in Kenya: *Otolemur g. lasiotis* (Peters, 1876), White-tailed Small-eared Galago recorded from the Kenyan coast; *Otolemur g. panganiensis* Matschie, 1905, Pangani Small-eared Galago from extreme CS Kenya; and *Otolemur g. kikuyuensis* (Lönnberg, 1912), Kikuyu Small-eared Galago from the Kenyan highlands E of the Eastern Rift Valley.

**Genus** ***Galago*** É. Geoffroy, 1796. Lesser Galagos

32. ***Galago senegalensis*** É. Geoffroy, 1796. English: Northern Lesser Galago. Swahili: Komba ya Senegali. Chinese: 北小婴猴. Recorded from Senegal to the Gulf of Aden and much of eastern Africa. In savanna, woodland, bushland, closed forest and riverine woodland (Nash et al., 2013). Three subspecies recognized in Kenya: *Galago s. senegalensis* É. Geoffroy, 1796, Senegal Lesser Galago recorded from Mt. Elgon, Kenya; *Galago s. braccatus* Elliot, 1907, Kenya Lesser Galago from NW, C and SE Kenya; and *Galago s. sotikae* Hollister, 1920, Uganda Lesser Galago from SW Kenya.
33. ***Galago gallarum*** Thomas, 1901. English: Somali Lesser Galago. Swahili: Komba Somali. Chinese: 索马里小婴猴. Recorded from S Ethiopia, NE Kenya and NE Somalia. In *Acacia-Commiphora* bushland and thickets. In Kenya, recorded from the coastal strip of NE Kenya to the lower Tana River (Butynski & De Jong, 2013).

**Genus** ***Paragalago*** Master et al., 2017. Dwarf Galagos

Previously placed within *Galagoides* A. Smith, 1833 (Dwarf Galagos) as *Galagoides cocos* but now moved to newly proposed genus *Paragalago* (Master et al., 2017).
34. ***Paragalago cocos*** (Heller, 1912). English: Kenya Coast Dwarf Galago. Swahili: Komba. Chinese: 肯尼亚海岸倭丛猴. Recorded from Kenya and NE Tanzania. In dry mixed coastal forests, thickets and flood-plain forests. In Kenya, recorded from coastal forests in Kenya as far as the lower Tana River forests (Butynski et al., 2006; Harcourt & Perkin, 2013b as *Galagoides cocos*).

**ORDER RODENTIA (Rodents–106 species)**

**Family SCIURIDAE**

**Genus** ***Heliosciurus*** Trouessart, 1880. Sun Squirrels

35. ***Heliosciurus gambianus*** (Ogilby, 1835). English: Gambian Sun Squirrel. Swahili: Kindi. Chinese: 太阳松鼠. Widespread from Senegal to southern Sudan, South Sudan and eastern Ethiopia, also in parts of Angola, DR Congo and Zambia. Wooded savannas (Happold D, 2013b). In Kenya, recorded from Lodwar and W of Lake Turkana.
36. ***Heliosciurus rufobrachium*** (Waterhouse, 1842). English: Red-legged Sun Squirrel. Swahili: Kindi. Chinese: 红腿太阳松鼠. Widespread in West and Central Africa from Senegal to Kenya and Uganda; In DR Congo, only N of the Zaire River. Lowland moist rainforests, secondary and plantation forests with large trees (Emmons, 2013a). In Kenya, recorded only from Mt. Elgon.
37. ***Heliosciurus undulatus*** (True, 1892). English: Zanj Sun Squirrel. Swahili: Kindi. Chinese: 小太阳松鼠. Recorded only in SE Kenya and NE Tanzania, including Mafia and Zanzibar islands. Coastal forests and riverine vegetation (Schennum & Thorington, 2013a).

**Genus** ***Paraxerus*** Forsyth Major, 1893. Bush Squirrels

38. ***Paraxerus flavovittis*** (Peters, 1852). English: Striped Bush Squirrel. Swahili: Kindi Vichaka. Chinese: 黄纹丛松鼠. Recorded from SE Kenya, eastern Tanzania, N Mozambique and SE Malawi. Savannas, forests and cultivations with hardwood trees with holes (Schennum & Thorington, 2013b). In Kenya, recorded along coast S of Mombasa (Msambweni).
39. ***Paraxerus ochraceus*** (Huet, 1880). English: Ochre Bush Squirrel. Swahili: Kindi Vichaka. Chinese: 赭丛松鼠. Widespread in Kenya and NE Tanzania, with a few records from S Somalia and S South Sudan. Wide variety of habitats, including mountain forests, riverine forests, coasal forests, thickets and urban gardens (Thorington & Schennum, 2013). In Kenya, recorded from W, E and C, including the Tana River and Nairobi.
40. ***Paraxerus palliatus*** (Peters, 1852). English: Red Bush Squirrel. Swahili: Kindi Vichaka. Chinese: 南非红丛松鼠. Recorded from coastal Somalia to extreme NE of South Africa, including parts of E-C Tanzania and along the Zambezi/Shire Rivers to Malawi. Coastal, dunes and riverine forests (Thorington et al., 2013). In Kenya, recorded in coastal habitats.
41. ***Protoxerus stangeri*** (Waterhouse, 1842). English: Forest Giant Squirrel. Swahili: Kindi. Chinese: 非洲巨松鼠. Widely distributed in West and Central Africa from Sierra Leone to Uganda and W Kenya, with outliers in S DR Congo and Angola. Rainforests and secondary forests in rainforest zones (Emmons, 2013b). In Kenya, recorded from Kakamega Forest and N and S Nandi Forests.

**Genus** ***Xerus*** Hemprich and Ehrenberg, 1833. Ground Squirrels

42. ***Xerus erythropus*** (E. Geoffroy, 1803). English: Striped Ground Squirrel. Swahili: Kindi. Chinese: 条纹地松鼠. Widely distributed in West and Central Africa S of the Sahara, from Senegal and Mauritania to eastern Sudan. Semi-deserts, savanna woodlands, clearings in rainforests, and cultivated fields (Waterman, 2013a). In Kenya, recorded in NW extending southwards in the Rift Valley. May be sympatric with *X. rutilans* in the Rift Valley (Kingdon, 1974b).
43. ***Xerus rutilus*** (Cretzschmar, 1828). English: Unstriped Ground Squirrel. Swahili: Kindi. Chinese: 赤地松鼠. Recorded from the Horn of Africa from coastal Sudan, E Ethiopia and Somalia to NE Tanzania. Dry, semi-arid areas including agricultural fields in Kenya (Waterman, 2013b). In Kenya, widely distributed in dry habitats.

**Family GLIRIDAE**

**Genus** ***Graphiurus*** Smuts, 1832. Dormice 

The taxonomy of dormice in Africa is controversial and species are difficult to identify.

44. ***Graphiurus kelleni*** (Reuvens, 1890). English: Kellen’s African Dormouse. Swahili: Panya. Chinese: 卡伦非洲睡鼠. Widespread in disjunct populations in West, East and southern Africa, and in the Horn of Africa. Woodland savannas, riverine woodlands and rocky areas (Holden, 2013a). In Kenya, widely distributed, except in the NE.
45. ***Graphiurus microtis*** (Noack, 1887). English: Noack’s African Dormouse. Swahili: Panya. Chinese: 小非洲睡鼠. Widely distributed in the eastern half of Africa from Sudan to South Africa, Botswana and Namibia. Woodland habitats (Holden, 2013b). In Kenya, recorded from the NW (Lotikipi), SW and E (Narok, Kajiado, Taita).
46. ***Graphiurus murinus*** (Desmarest, 1822). English: Forest African Dormouse. Swahili: Panya. Chinese: 非洲林睡鼠. Widely distributed in the eastern half of Africa from Ethiopia to South Africa. Afroalpine, riverine and coastal forests (Holden, 2013c). In Kenya, widespread in western half and in the SE, including Mt. Gargues, Mathews Range, Mt. Nyiru, Marsabit and Karissia Hills.

**Family SPALACIDAE**

**Genus** ***Tachyoryctes*** Ruppell, 1835. Root-rats

The taxonomy of this genus is complex and not yet resolved. Musser & Carleton (2005) considered *T. ankoliae, T. annectens, T. audax, T. daemon, T. ibeanus, T. naivashae, T. rex, T. ruandae, T. ruddi, T. spalacinus* and *T. storeyi* as valid species in Kenya, whereas Jarvis (2013a) considered these as synonyms of *T. splendens*. We have followed Monadjem et al. (2015) who considered *T. rex, T. annectens, T. ibeanus, T. spalacinus* and *T. ruddi* as valid species in Kenya based on morphometric analysis and the distinct biogeographic and ecological distributions of each species.

47. ***Tachyoryctes annectens*** (Thomas, 1981). English: Mianzini Root-rat. Swahili: Fuko/Mizizi Panya. Chinese: 美兹尼鼹鼠. Included within *T. splendens* by Jarvis (2013a). Endemic to Kenya. Subterranean in well-drained soils in savanna habitats. In Kenya, recorded at Mianzini and E of Lake Naivasha (Musser & Carleton, 2005).
48. ***Tachyoryctes ibeanus*** Thomas, 1900. English: Kenyan Root-rat. Swahili: Fuko/Mizizi Panya. Chinese: 肯尼亚鼹鼠. Included within *T. splendens* by Jarvis (2013a). Musser & Carleton (2005) recognized the taxa *T. storey* and *T. naivashae* as specifically distinct from *T. ibeanus*. However, based on skull morphometrics and biogeography, Monadjem et al. (2015) considered these three taxa to be conspecific. Thus, pending molecular studies, we have treated these three taxa as conspecific. Endemic to Kenya. Subterranean in well-drained soils in savanna habitats. In Kenya, recorded near Nairobi and on the western part of the Athi Plains (Musser & Carleton, 2005; Monadjem et al., 2015).
49. ***Tachyoryctes rex*** Heller, 1910. English: King Root-rat. Swahili: Fuko/Mizizi Panya. Chinese: 小鼹鼠. Included within *T. splendens* by Jarvis (2013a). Endemic to Kenya. In montane and alpine habitats. In Kenya, recorded only on the higher slopes of Mt. Kenya (ca. 2 600–3 500 m a.s.l.) (Musser & Carleton, 2005).
50. ***Tachyoryctes ruddi*** Thomas, 1909. English: Rudd’s African Root-rat. Swahili: Fuko/Mizizi Panya. Chinese: 拉德鼹鼠. Included within *T. splendens* by Jarvis (2013a). Recorded in a small area of W Kenya, SW Uganda and NW Tanzania. In tropical rainforests and montane forests. In Kenya, recorded in Kakamega and the lower slopes of Mt. Elgon (Monadjem et al., 2015, Musser & Carleton, 2005).
51. ***Tachyoryctes spalacinus*** Thomas, 1909. English: Embi African Root-rat. Swahili: Fuko/Mizizi Panya. Chinese: 高山鼹鼠. Included within *T. splendens* by Jarvis (2013a). Endemic to Kenya. In montane forests. In Kenya, recorded on the lower slopes of Mt. Kenya and on the plains and foothills S and W of Mt. Kenya (Monadjem et al., 2015, Musser & Carleton, 2005).

**Family NESOMYIDAE**

**Genus** ***Beamys*** Thomas, 1909. Long-tailed Pouched Rats

52. ***Beamys hindei*** Thomas, 1909. English: Long-tailed Pouched Rat. Swahili: unavailable. Chinese: 长尾巨鼠. Recorded in scattered localities in Kenya, Tanzania, Malawi and Zambia. Evergreen and slightly deciduous forests and riverine forests close to streams. In Kenya, recorded S of Mombasa and in the Arabuko-Sokoke Forest (Happold D, 2013c).

**Genus** ***Cricetomys*** Waterhouse, 1840. Giant Pouched Rats

53. ***Cricetomys ansorgei*** Thomas, 1904. English: Southern Giant Pouched Rat. Swahili: Panya Buku. Chinese: 非洲巨鼠. Previously included within *C. gambianus* (Duplantier, 2013), but shown to be specifically distinct (Olayemi et al., 2012). Widely distributed in southern and eastern Africa from SW Kenya and northern Tanzania to S DR Congo, Angola, Zambia, Malawi, Mozambique, Zimbabwe and South Africa (Musser & Carleton, 2005), although western limits are not yet known may extend into Uganda. Forests, savanna and human-modified habitats. Widespread in W and SE Kenya (Monadjem et al., 2015).

**Genus** ***Saccostomus*** Peters, 1846. Pouched Mice

54. ***Saccostomus mearnsi*** Heller, 1910. English: Mearns’ Pouched Mouse. Swahili: unavailable. Chinese: 东岸囊鼠. Recorded from SW Ethiopia to Kenya, S Somalia, E Uganda and NE Tanzania (Keesing, 2013, Mikula et al., 2016). Savanna woodlands. In Kenya, widely distributed, except in parts of the SE (Keesing, 2013).
55. ***Saccostomus umbriventer*** Miller, 1910. English: Brown-bellied Pouched Mouse. Swahili: unavailable. Chinese: 褐腹囊鼠. Included within *S. mearnsi* by Musser & Carleton (2005) and Keesing (2013). Recorded only from a narrow region in N Tanzania and SW Kenya (Mikula et al., 2016). Dry savanna habitats. In Kenya, dry savanna habitats in the SW.

**Genus** ***Dendromus*** Smith, 1829. Climbing Mice

56. ***Dendromus insignis*** (Thomas, 1903). English: Montane African Climbing Mouse. Swahili: Panya. Chinese: 异攀鼠. Recorded from a few small and scattered populations in W DR Congo, Uganda, Kenya and Tanzania. Grassy patches, marshes and moist herbaceous vegetation in montane and highland habitats. In Kenya, recorded from the Mathews Range, Mt. Kenya, Aberdare Ranges, Mau Escarpment and Cherangani Hills (Dieterlen, 2013a).
57. ***Dendromus melanotis*** Smith, 1834. English: Grey African Climbing Mouse. Swahili: Panya. Chinese: 黑背攀鼠. Widely distributed in southern Africa, with small outlier populations in Ethiopia, Liberia, Togo, Nigeria, Uganda, Kenya and Tanzania. Wide range of habitats from grasslands to woodlands (Monadjem, 2013a; Monadjem et al., 2015). In Kenya, restricted to the S and W.
58. ***Dendromus messorius*** (Thomas, 1903). English: Banana African Climbing Mouse. Swahili: Panya. Chinese: 汤氏攀鼠. Recorded from very small and highly scattered populations in Ghana, Togo, Cameroon, NE DR Congo, Uganda and Kenya. Forested areas and grasslands, as well as banana plantations and cultivated areas (Happold D, 2013d). In Kenya, recorded from Mt. Elgon (as *D. mysticalis ruddi*-see Musser and Carleton, 2005)
59. ***Dendromus mystacalis*** Heuglin, 1863. English: Chestnut African Climbing Mouse. Swahili: Panya. Chinese: 须攀鼠. Recorded in many countries on the eastern side of Africa, from Ethiopia to South Africa. Grassland and savanna habitats. In Kenya, recorded in the SE (Monadjem, 2013b).

**Genus** ***Steatomys*** Peters, 1846. Fat Mice

60. ***Steatomys parvus*** Rhoads, 1896. English: Tiny African Fat Mouse. Swahili: Panya. Chinese: 矮肥鼠. Disjunct and separate distributions in Zambia, Botswana, Angola and eastern Africa. (Monadjem, 2013c). Dry grasslands, woodlands and open scrublands. In Kenya, recorded in the S (Monadjem, 2013c).

**Family CRICETIDAE**

**Genus** ***Lophiomys*** Milne-Edwards, 1867. Maned Rats

61. ***Lophiomys imhausi*** Milne-Edwards, 1867. English: Maned Rat. Swahili: Panya. Chinese: 东非冠鼠. Disjunct distribution in E Sudan, Djibouti, Ethiopia, Somalia, Uganda and Kenya. Recorded in rocky areas and dry woodlands, but also in moist and montane forests in Kenya (Happold D, 2013e). In Kenya, recorded from C and W regions, especially the Central Highlands and on Mt. Elgon (Kingdon, 1974b).

**Family MURIDAE**

**Genus** ***Acomys*** I. Geoffroy, 1838. Spiny Mice

62. ***Acomys cineraceus*** Heuglin and Fitzinger, 1866. English: Grey Spiny Mouse. Swahili: Panya. Chinese: 灰刺毛鼠. Recorded in Sudan, South Sudan, Ethiopia and N Kenya. Dry rocky habitats and semi-arid areas. In Kenya, recorded from the W and E of Lake Turkana (Dieterlen, 2013b).
63. ***Acomys ignitus*** Dollman, 1910. English: Fiery Spiny Mouse. Swahili: Panya. Chinese: 焰刺毛鼠. Endemic to SE Kenya and extreme NE Tanzania. Rocky habitats in dry savanna grasslands. In Kenya, known from Voi and Tsavo NP (Dieterlen, 2013c).
64. ***Acomys kempi*** Dollman, 1911. English: Kemp’s Spiny Mouse. Swahili: Panya. Chinese: 肯氏刺毛鼠. Recorded from Kenya, S Ethiopia, S Somalia and extreme NE Tanzania. Rocky habitats in dry savanna and semi-desert. In Kenya, widely distributed in dry areas E of the Rift Valley (Dieterlen, 2013d).
65. ***Acomys percivali*** Dollman, 1911. English: Percival’s Spiny Mouse. Swahili: Panya. Chinese: 佩氏刺毛鼠. Recorded in small areas of S Sudan, SW Ethiopia, N Uganda and NE Kenya (extending southwards along the Rift Valley). Rocky habitats. In Kenya, widespread in the NW (e.g., Chandler’s Falls-Nyiro) and in the Rift Valley (Takata, 2013a).
66. ***Acomys wilsoni*** Thomas, 1892. English: Wilson’s Spiny Mouse. Swahili: Panya. Chinese: 威氏刺毛鼠. Recorded in South Sudan, S Ethiopia and Somalia, Kenya and N Tanzania. Rocky habitats and grasslands with shrubs. In Kenya, widely distributed in most of the country, except W to C Kenya (Takata, 2013b).

**Genus** ***Lophuromys*** Peters, 1874. Brush-furred Rats

The taxonomy of the genus is controversial (Dieterlen, 2013e) and has not yet been resolved. Following Musser & Carleton (2005) and Monadjem et al. (2015), we recognize three species here.

67. ***Lophuromys ansorgei*** de Winton, 1986. English: Ansorge’s Brush-furred Rat. Swahili: Panya. Chinese: 安氏刚毛鼠. Included within *L. sikapusi* by Dieterlen (2013f). Recorded from E DR Congo (close to Zaire River), W DR Congo, Rwanda, W Uganda and Kenya. Widely distributed in rainforests and montane forests. In Kenya, recorded from Nyanza close to Lake Victoria (Monadjem et al., 2015).
68. ***Lophuromys margarettae*** Heller, 1912. English: Margaretta’s Brush-furred Rat. Swahili: Panya. Chinese: 马氏刚毛鼠. Included within *L. flavopunctatus* by Dieterlen (2013g). Recorded in Kenya, Uganda and southern South Sudan. Highland forests and grasslands. In Kenya, recorded widely in the southern highlands, including Mt. Kenya (lower elevations) and Aberdare Ranges (Monadjem et al., 2015).
69. ***Lophuromys zena*** Dollman, 1909. English: Zena’s Brush-furred Rat. Swahili: Panya. Chinese: 泽娜刚毛鼠. Included within *L. flavopunctatus* by Dieterlen (2013g). In Kenya, it is sympatric with *L. margarettae* on Mt. Kenya and the Aberdare Ranges (Monadjem et al., 2015), but typically occurs at higher elevations than the latter species (Verheyen et al., 2007).

**Genus** ***Uranomys*** Dollman, 1909. Brush-furred Rats

70. ***Uranomys ruddi*** Dollman, 1909. English: Rudd’s Brush-furred Rat. Swahili: Panya. Chinese: 白腹蓬毛鼠. Widely distributed in West Africa, but also discrete populations in Central, East and southern Africa. Moist savannas, grasslands and oil plantations (in West Africa) (Happold D, 2013f). The presence of this species in Kenya is only known by the type specimen from “Kirui, southern foothills of Mt. Elgon, Kenya” (Delany, 1975). Due to possible confusion regarding the exact locality mentioned in Dollman (1909), the presence of this species in Kenya requires confirmation.

**Genus** ***Gerbilliscus*** Thomas, 1897. Gerbils

71. ***Gerbilliscus boehmi*** (Noack, 1887). English: Boehm’s Gerbil. Swahili: Panya. Chinese: 波氏大沙鼠. Formerly placed in the genus *Tatera.* Recorded from S Uganda, Rwanda, Burundi, W Tanzania, Malawi, W Mozambique, NW Zambia, S DR Congo and E Angola, with isolated populations in S Kenya. *Brachystegia* woodlands with good grass and herb cover (Happold D, 2013g). In Kenya, recorded from areas near the Lower Ewaso Ng’iro River in the SW (Musser & Carleton, 2005).
72. ***Gerbilliscus kempi*** (Wroughton, 1906). English: Kemp’s Gerbil. Swahili: Panya. Chinese: 凯氏大沙鼠. Previously *Tatera kempi*, but now placed in the genus *Gerbilliscus*. Recorded from Gambia and Sierra Leone eastwards to S Sudan, N DR Congo, Uganda, Kenya, Rwanda and Burundi. Savanna grasslands with good grass and shrub cover, as well as abandoned farmlands (Happold D, 2013h). In Kenya, recorded in the NE.
73. ***Gerbilliscus nigricaudus*** (Peters, 1878). English: Black-tailed Gerbil. Swahili: Panya. Chinese: 黑尾沙鼠. Formerly placed in the genus *Tatera*. Recorded from S Ethiopia, S Somalia, Kenya and NE Tanzania. Dry savanna woodlands and grasslands (Happold D, 2013i). In Kenya, widespread, although distribution is patchy.
74. ***Gerbilliscus phillipsi*** (de Winton, 1898). English: Phillips’s Gerbil. Swahili: Panya. Chinese: 菲利普大沙鼠. Formerly placed in the genus *Tatera*. Recorded disjunctively in C and S Ethiopia, Somaliland and Kenya. Dry arid savannas and semi-deserts (Happold D, 2013j). In Kenya, known only from near Baringo in the Rift Valley.
75. ***Gerbilliscus vicinus*** (Peters, 1878). English: Vicinus Gerbil. Swahili: Panya. Chinese: 维奇尼大沙鼠. Formerly placed in the genus *Tatera*; included within *Gerbilliscus robustus* by Happold D (2013k). Recorded from Tanzania and Kenya. Dryland areas (Monadjem et al., 2015). In Kenya, found widely throughout the country but appears to be absent from the drier regions in the NE and highlands of the SW.

**Genus** ***Gerbillus*** Desmarest, 1804 Gerbils

76. ***Gerbillus cosensi*** Dollmann, 2014 English: Cosen’s Gerbil. Swahili: Panya. Chinese: 饰小沙鼠. Sometimes considered as a synonym of *G. agag* (Musser & Carleton, 2005). Endemic to NE Uganda and NW Kenya. Dry semi-arid habitats. In Kenya, recorded in the region of Lake Turkana (Turkwel Valley, Lodwar, Lokori) and Archer’s Post (Happold D, 2013l).
77. ***Gerbillus harwoodi*** Thomas, 1901. English: Harwood’s Gerbil. Swahili: Panya. Chinese 哈伍德小沙鼠. Recorded from Kenya and N-C Tanzania. Grasslands in *Acacia-*savanna (Happold D, 2013m). In Kenya, recorded from the highlands and the Rift Valley in the S.
78. ***Gerbillus pusillus*** Peters, 1878. English: Least Gerbil. Swahili: Panya. Chinese; 肯尼亚小沙鼠. Small isolated population. Recorded disjunctively from C Ethiopia, South Sudan, Somalia, Kenya and N Tanzania. Short dry grasslands on sandy soils. In Kenya, recorded from near Lake Turkana and in the SE (Happold D, 2013n).

**Genus** ***Taterillus*** Thomas, 1910. Taterils

79. ***Taterillus emini*** (Thomas, 1892). English: Emin’s Tateril. Swahili: Panya. Chinese: 乍得小裸掌沙鼠. Recorded in Sudan, South Sudan, Ethiopia, Somalia, NE DR Congo. Kenya and N Tanzania. Dry and moist savanna habitats (Granjon & Dobigny, 2013). In Kenya, widely distributed in suitable habitats.

**Genus** ***Aethomys*** Thomas, 1915. Veld Rats

80. ***Aethomys chrysophilus*** (de Winton, 1897). English: Red Veld Rat. Swahili: Panya. Chinese: 金毛蹊鼠. Recorded from Kenya southwards to Angola, Namibia, Botswana, Mozambique and N South Africa. Savannas woodlands. Separate population ranges occur in N and SE Kenya (Linzey et al., 2013a).
81. ***Aethomys hindei*** (Thomas, 1902). English: Hinde’s Veld Rat. Swahili: Panya. Chinese: 中非蹊鼠. Widely distributed in Central and East Africa in rocky areas, dense grass and bush cover, moist and disturbed habitats (Linzey et al., 2013b). In Kenya, recorded from south of Lake Turkana and in the coastal SE.
82. ***Aethomys kaiseri*** (Noack, 1887). English: Kaiser’s Veld Rat. Swahili: Panya. Chinese: 凯氏蹊鼠. Recorded from E Angola eastwards to Uganda, Kenya and Tanzania. Savanna habitats with trees and shrubs. In Kenya, restricted to a narrow band in the S along the Kenya-Tanzania border (Linzey et al., 2013c).

**Genus** ***Arvicanthis*** Lesson, 1842. Grass Rats

83. ***Arvicanthis nairobae*** J.A. Allen, 1909. English: Nairobi Grass Rat. Swahili: Panya. Chinese: 肯尼亚垄鼠. Recorded in S-C Kenya and N-C Tanzania. Grasslands and savanna habitats, mostly in highlands, especially where habitat is dense (Takata, 2013c). In Kenya, recorded mostly in highlands east of the Rift Valley.
84. ***Arvicanthis neumanni*** (Matschie 1894). English: Neumann’s Grass Rat. Swahili: Panya. Chinese: 诺伊曼垄鼠. Recorded in E Ethiopia, Somalia, Kenya and N-C Tanzania. Recorded in dry bush and savanna habitats (Bekele, 2013). In Kenya, found only in the NE (Mandera, Marsabit) and extreme SE.
85. ***Arvicanthis niloticus*** (É. Geoffroy, 1803). English: Nile Grass Rat. Swahili: Panya. Chinese: 尼罗垄鼠. Widely distributed from Senegal to E Sudan and Ethiopia, extending southwards in eastern Africa to Tanzania and Zambia. Savanna grasslands near water sources, shrublands and cultivations (Granjon et al., 2013). In Kenya, recorded in western half of the country.

**Genus** ***Colomys*** Thomas and Wroughton, 1907. African Water Rat

86. ***Colomys goslingi*** Thomas & Wroughton, 1907. English: African Water Rat. Swahili: Panya. Chinese: 居鼠. Recorded disjunctively in Cameroon, DR Congo (N of Zaire River), Burundi, Uganda and Kenya, with isolated populations also in Angola and Zambia. Streams and waterways in rainforest and montane forest riverine habitats (Dieterlen, 2013h). In Kenya, recorded in the SW highland areas, including Kakamega Forest and Mt. Elgon.

**Genus** ***Dasymys*** Peters, 1875. Shaggy Rats

87. ***Dasymys incomtus*** (Sundevall, 1847). English: Common Shaggy Rat. Swahili Panya. Chinese: 非洲水鼠. Widely distributed in eastern and southern Africa, as well as Ethiopia, Sudan and South Sudan. Reed-beds, long grass close to water, and damp areas on drainage lines (Pillay, 2013). In Kenya, widely distributed in the W and S, mostly west of the Rift Valley.

**Genus** ***Grammonys*** Thomas, 1915. Thickets Rats

88. ***Grammomys brevirostris*** Krystufek, 2008. English: Short-snouted Thicket Rat. Swahili: Panya. Chinese: 短吻线鼠. Endemic to Kenya. Savanna grasslands (Krystufek 2008). In Kenya, only known from type locality (Lemesikio, Loliondo, Loita plains).
89. ***Grammomys caniceps*** Hutterer & Dieterlen, 1984. English: Gray-headed Thicket Rat. Swahili: Panya. Chinese: 灰头线鼠. Recorded from Somalia and Kenya, only along coastal regions. Dry coastal savanna with trees and palms (Hutterer, 2013a). In Kenya, recorded from the coast N of Mombasa (Malindi).
90. ***Grammomys dolichurus*** (Smuts, 1832). English: Common Thicket Rat. Swahili: Panya. Chinese: 南非线鼠. Recorded widely in the eastern half of Africa from Uganda and Kenya to South Africa, extending westwards S of the Congo basin to Angola. Woodland savanna and gallery forests (Happold D, 2013o). In Kenya, recorded from the SW, mostly W of the Rift Valley, with a narrow extension to the coast in the extreme SE.
91. ***Grammomys gigas*** Dollman, 1911. English: Giant Thicket Rat. Swahili: Panya. Chinese. 巨线鼠. Endemic to Kenya. Afro-alpine habitat. In Kenya, recorded only from the type locality at Solai, Mt. Kenya (2 740 m a.s.l.) (Dieterlen, 2013i).
92. ***Grammomys ibeanus*** (Osgood, 1910). English: East African Thicket Rat. Swahili: Panya. Chinese: 莫洛线鼠. Recorded from disjunct small areas from South Sudan, Uganda, Kenya, Tanzania and Malawi. Evergreen montane forests and dense thickets (Dieterlen, 2013j). In Kenya, recorded from Mt. Elgon, Mt. Gargues, Mathews Range, Karissia Hills and Mt. Nyiru and along the escarpments of the Rift Valley.
93. ***Grammomys macmillani*** (Wroughton, 1907). English: Macmillan’s Thicket Rat. Swahili: unavailable. Chinese: 马氏线鼠. Recorded from small and scattered areas in Sierra Leone, Central African Republic, South Sudan, Kenya, Uganda, S Ethiopia and Tanzania. Forests, riverine forests, grasslands and undergrowth (Dieterlen, 2013k). In Kenya, recorded only in the extreme SE (Msambweni and Lunga Lunga).

**Genus** ***Hylomyscus*** Thomas, 1926. Wood Mice

94. ***Hylomyscus endorobae*** (Thomas, 1906). English: Endorobo Wood Mouse. Swahili: Panya. Chinese: 高山柔毛鼠. Placed within *Hylomyscus denniae* by Dieterlen (2013l). Endemic to Kenya. Afro-montane forests. In Kenya, recorded from upper and lower slopes of Mt. Kenya, Aberdare Ranges and Mau Escarpment (Carleton & Byrne, 2006). Specimens from Mt Elgon (as *H. denniae*; Clausnitzer, 2003) and Cherangani Hills may belong to this species.
95. ***Hylomyscus kerbispeterhansi*** Demos, Agwanda & Hickerson, 2014. English: Kerbispeterhans’s Wood Mouse. Swahili: Panya. Chinese: 克氏柔毛鼠. Endemic to Kenya. Montane habitats. In Kenya, recorded from Aberdare Ranges, Cherangani Hills and Mt. Elgon (Demos et al., 2014).
96. ***Hylomyscus kaimosae*** (Heller, 1912). English: Kaimosi Wood Mouse. Swahili: Panya. Chinese: 凯莫斯柔毛鼠. Placed within *Hylomyscus stella* by Dieterlen (2013m). Recorded from isolated populations in W Kenya, C Tanzania and S South Sudan. Montane and upland forests. In Kenya, recorded in the Kakamega Forest and other forests near Lake Victoria (Dieterlen, 2013m, as *Hylomyscus stella*).

**Genus** ***Lemniscomys*** Trouessart, 1881. Grass Mice

97. ***Lemniscomys macculus*** (Thomas & Wroughton, 1910). English: Buffoon Grass Mouse. Swahili: Panya. Chinese: 花草鼠. Recorded in NE DR Congo, and parts of Uganda, SE Sudan, N Kenya and SW Ethiopia. Open grasslands with *Acacia* trees and *Euphorbia candelabra*, rocky areas and dry river beds (Dieterlen, 2013n). In Kenya, recorded from some parts of the NE.
98. ***Lemniscomys rosalia*** (Thomas, 1904). English: Single-striped Grass Mouse. Swahili: Panya. Chinese: 蔷薇草鼠. Recorded widely in southern Africa and small areas of Kenya and Tanzania in savanna habitats and cultivated areas (Monadjem, 2013d). In Kenya, recorded in the SE, S of Mombasa.
99. ***Lemniscomys striatus*** (Linnaeus, 1758). English: Striated Grass Mouse. Swahili: Panya. Chinese: 斑草鼠. Recorded from Sierra Leone to eastern Africa and C Ethiopia, and in S DR Congo, N Zambia and N Angola. Grasslands, woodland savanna, farmlands and open grassy areas in rainforest (Happold D, 2013p). In Kenya, recorded widely in the W.
100. ***Lemniscomys zebra*** (Heuglin, 1864). English: Zebra Grass Mouse. Swahili: Panya. Chinese: 休氏草鼠. Recorded from Senegal to S Sudan, South Sudan, Uganda, Kenya and Tanzania. Dry grasslands and wooded grasslands with low rainfall. In Kenya, recorded in the W and S to W of the Rift Valley (Happold D & Dieterlen, 2013).

**Genus** ***Mastomys*** Thomas, 1915. Multimammate Mice

101. ***Mastomys erythroleucus*** (Temminck, 1853). English: Guinea Multimammate Mouse. Swahili: Panya. Chinese: 红乳鼠. Recorded over a wide area from Senegal and Mauretania to Uganda, Kenya, Tanzania and Ethiopia. Grasslands, secondary forests, agricultural fields and foodstores (Leirs, 2013a). In Kenya, recorded in the C and NW.
102. ***Mastomys natalensis*** (Smith, 1834). English: Natal Multimammate Mouse. Swahili: Panya. Chinese: 南非乳鼠. Recorded over most of sub-Saharan Africa, except the extreme SW of the continent (parts of Namibia, Botswana and South Africa). Grasslands, wooded savannas, fields, thickets and human-modified habitats (Leirs, 2013b). In Kenya, recorded throughout most of country.
103. ***Mastomys pernanus*** (Kershaw, 1921). English: Dwarf Multimammate Mouse. Swahili: Panya. Chinese: 倭乳鼠. Recorded from small areas of N Tanzania and S Kenya. *Brachystegia* woodlands (Leirs, 2013c). In Kenya, recorded from the extreme SW (Mara River region).

**Genus** ***Mus*** Linnaeus, 1758. Old World Mice and Pygmy Mice

104. ***Mus mahomet*** Rhoads, 1896. English: Mahomet Pygmy Mouse. Swahili: Panya. Chinese: 索马里小家鼠. Recorded in Eritrea and Ethiopia, and perhaps in SW Kenya and SW Uganda (status uncertain). Montane forests, scrublands and grasslands (Ethiopia) (Yalden, 2013a). In Kenya, presence uncertain, with no locality records currently available.
105. ***Mus minutoides*** Smith, 1834. English: Tiny Pygmy Mouse. Swahili: Panya. Chinese: 南非小家鼠. Based on molecular characterization, this species has been recorded widely throughout sub-Saharan Africa, including West Africa (see Monadjem et al., 2015). The similar and closely related *Mus musculoides* (West African Pygmy Mouse) is widely recorded from West Africa, where it is sympatric with *M. minutoides*. Hence, recent molecular studies have clarified some of the confusion raised and discussed in the *Mammals of Africa* accounts (Happold D, 2013q; Monadjem, 2013e). Savanna woodlands, grasslands, rocky areas, broad-leaved woodlands and farmlands. In Kenya, recorded in the NW and S.
106. ***Mus musculus*** Linnaeus, 1758. English: House Mouse. Swahili: Panya. Chinese: 小家鼠. Exotic species in Africa. Recorded in many well-separated locations on the continent and inland in some places. Human habitations and some human-modified environments (Happold D, 2013r). In Kenya, recorded from urban centers (e.g., Nairobi).
107. ***Mus sorella*** (Thomas, 1909). English: Thomas’s Pygmy Mouse. Swahili: Panya. Chinese: 乌干达小家鼠. Recorded in a few discrete areas of the E DR Congo, Uganda, Kenya and Tanzania. Savanna grasslands and woodlands close to gallery forests (Petter, 2013a). In Kenya, recorded only on Mt. Elgon.
108. ***Mus tenellus*** (Thomas, 1903). English: Delicate Pygmy Mouse. Swahili: Panya. Chinese: 娇小家鼠. Recorded mainly from Ethiopia, with isolated populations in C Sudan, South Sudan, Kenya and Tanzania. Grasslands with thicket clumps (Petter, 2013b). In Kenya, single records in C and S regions.
109. ***Mus triton*** (Thomas, 1909). English: Gray-bellied Pygmy Mouse. Swahili: Panya. Chinese: 海神小家鼠. Recorded from South Sudan, Uganda, Kenya, Tanzania, E DR Congo, Malawi and Zambia. Grasslands with dense cover, forest edges and cultivations, especially in montane regions (Dieterlen & Happold D, 2013). In Kenya, recorded in the S and W.

**Genus** ***Mylomys*** Thomas, 1906. Three-toed Grass Rat

110. ***Mylomys dybowskii*** (Pousargues, 1893). English: Dybowski’s Three-toed Grass Rat. Swahili: Panya. Chinese: 非洲沟齿鼠. Recorded disjunctly from West Africa to Kenya. Rainforest-savanna mosaics and forest edges (Dieterlen, 2013o). In Kenya, recorded in the C and W.

**Genus** ***Myomyscus*** Shortridge, 1942. Meadow Mice

111. ***Myomyscus brockmani*** (Thomas, 1908). English: Brockman’s Meadow Mouse. Swahili: Panya. Chinese: 布氏软毛鼠. Recorded in E DR Congo, South Sudan, Uganda, Kenya and Tanzania, with isolated populations in W Sudan, Central African Republic, SW Ethiopia and NW Somalia. Rocky habitats and boulders in high altitude areas (Happold D, 2013s). In Kenya, widely distributed W of the Rift Valley.

**Genus** ***Oenomys*** Thomas, 1904. Rufous-nosed Rats

112. ***Oenomys hypoxanthus*** (Pucheran, 1855). English: Common Rufous-nosed Rat. Swahili: Panya. Chinese: 褐鼻鼠. Recorded widely from Nigeria, Cameroon and Gabon to DR Congo and East Africa, with isolated populations in W Ethiopia, Angola and Tanzania. Moist dense grasslands, forest edges, montane habitats and cultivated areas (Dieterlen, 2013p). In Kenya, recorded in the W and C (Aberdare Ranges).

**Genus** ***Otomys*** F. Curvier, 1824. Vlei Rats

The number of species in this genus increased dramatically with recent molecular studies (see Monadjem et al., 2015). Taylor (2013a) recognized 15 species in Africa, which was increased to 31 species by Monadjem et al. (2015). Of these, eight species have been recorded in Kenya.

113. ***Otomys angoniensis*** Wroughton, 1906. English: Angoni Vlei Rat. Swahili: Panya. Chinese: 阿贡沼鼠. Recorded from Kenya to South Africa, including Angola and Zambia. Mesic grasslands and savanna woodland habitats near swamps and water (Taylor, 2013b). In Kenya, recorded widely in the S and W at higher elevations.
114. ***Otomys barbouri*** Lawrence & Loveridge, 1953. English: Barbour’s Vlei Rat. Swahili: Panya. Chinese: 巴氏沼鼠. Endemic to Kenya and Uganda. Alpine heath on upper slopes (above 3 200 m a.s.l.). In Kenya, recorded only on Mt. Elgon (Clausnitzer, 2013).
115. ***Otomys dollmani*** Heller, 1912. English: Dollman’s Vlei Rat. Swahili: Panya. Chinese: 道氏沼鼠. Included within *O. tropicalis* by Taylor (2013c). In the past, placed as a subspecies of *O. irroratus* or *O. tropicalis* but considered as a valid species (Carleton & Byrne, 2006). Endemic to Kenya. Highland forests. In Kenya, recorded only from Mount Gargues (Urguess) in the Mathews Range.
116. ***Otomys jacksoni*** Thomas, 1891. English: Jackson’s Vlei Rat. Swahili: Panya. Chinese: 杰氏沼鼠. Included within *O. typus* by Yalden (2013b) but as a valid species by Musser & Carleton (2005). Endemic to Kenya and Uganda. Alpine habitats (3 300–4 200 m a.s.l.). In Kenya, recorded only on Mt. Elgon (Monadjem et al., 2015).
117. ***Otomys orestes*** Thomas, 1900. English: Afroalpine Vlei Rat. Swahili: Panya. Chinese: 非洲高山沼鼠. Formerly considered a synonym of *O. irroratus*, *O. tropicalis* or *O. typus* but now considered as a valid species (Carleton & Byrne, 2006). Endemic to Kenya in alpine habitats. In Kenya, recorded only on Mt. Kenya and the Aberdare Ranges (Musser & Carleton, 2005; Taylor et al., 2011).
118. ***Otomys thomasi*** Osgood, 1910. English: Thomas’ Vlei Rat. Swahili: Panya. Chinese: 托氏沼鼠. Endemic to Kenya. Afro-alpine grasslands, scrub and heathland at higher altitudes (Monadjem et al., 2015). In Kenya, recorded from higher elevations of the Mau Escarpment (2 450–2700 m a.s.l.) and the Uasin Gishu Plateau W of the Rift Valley (Monadjem et al., 2015).
119. ***Otomys tropicalis*** Thomas, 1902. English: Tropical Vlei Rat. Swahili: Panya. Chinese: 热带沼鼠. Recorded in scattered localities in South Sudan, E DR Congo, Uganda, Burundi, Uganda and Kenya. Afro-alpine grasslands, scrub and heathland at higher elevations (Taylor, 2013c). In Kenya, recorded widely from Mt. Elgon to the Kenyan Rift (Monadjem et al., 2015).
120. ***Otomys typus*** (Heuglin, 1877). English: Ethiopian Vlei Rat. Swahili: Panya. Chinese: 横沼鼠. Recorded from N and C Ethiopia, with small disjunct populations in Kenya, Uganda, Tanzania and N Malawi. Moist grasslands and swamps at higher elevations. In Kenya, recorded on Mt. Elgon (Yalden, 2013b).

**Genus** ***Pelomys*** Peters, 1852. Creek Rats

121. ***Pelomys fallax*** (Peters, 1852). English: East African Creek Rat. Swahili: Panya. Chinese: 沟齿泽鼠. Recorded from N Angola and DR Congo to Uganda, Kenya and Tanzania, and south to Zambia, Malawi and Mozambique. Creeks, savannas with thick moist grass, swamps and where water is available for most of the year (Dieterlen, 2013q). In Kenya, recorded from the S, close to the Kenya-Tanzania border.
122. ***Pelomys hopkinsi*** Hayman, 1955. English: Hopkins’s Creek Rat. Swahili: Panya. Chinese: 卢安达泽鼠. Recorded in small and isolated areas around Lake Victoria (SW Uganda, C and S Rwanda and W Kenya). Occurs in papyrus swamp areas. In Kenya, restricted to papyrus swampy areas around Lake Victoria (Dieterlen, 2013r).

**Genus** ***Praomys*** Thomas, 1915. Soft-furred Mice

The taxonomy of the genus *Praomys* is controversial and has been the subject of debate for many years. The definition of the genus and constituent species are still uncertain (Happold D, 2013t). For Africa as a whole, 16 species are recognized (Happold D, 2013t), with three species occurring in Kenya.

123. ***Praomys delectorum*** (Thomas, 1910). English: East African Soft-furred Mouse. Swahili: Panya. Chinese: 赤道柔毛鼠. Recorded in Kenya, Tanzania, Malawi and Mozambique. Montane forests in isolated highland regions (Happold D, 2013u). In Kenya, recorded from the Shimba Hills.
124. ***Praomys jacksoni*** (de Winton, 1897). English: Jackson’s Soft-furred Mouse. Swahili: Panya. Chinese: 杰氏柔毛鼠. Recorded from Nigeria to eastern East Africa, including parts of South Sudan, Zambia, Uganda and Kenya. Rainforests, lowland montane forests and secondary forests (Dieterlen, 2013s). In Kenya, recorded in a few localities in the W.
125. ***Praomys misonnei*** Van der Straeten and Dieterlen, 1987. English: Misonne’s Soft-furred Mouse. Swahili: Panya. Chinese: 米氏柔毛鼠. Widespread from the Volta River, Central Africa, N to W DRC and parts of East Africa in lowland rainforests. In Kenya, recorded from the W to Kakamega Forest (Monadjem et al., 2015).

**Genus** ***Rattus*** Fischer, 1803. Rats

126. ***Rattus rattus*** (Linnaeus, 1758). English: Black Rat. Swahili: Panya. Chinese: 黑家鼠. Exotic species in Africa. Widespread in coastlines and inland within large urban areas and cities, especially those close to railway lines (Happold D, 2013v). In Kenya, restricted to the S and W.

**Genus** ***Rhabdomys*** Thomas, 1916. Four-striped Grass Mice

127. ***Rhabdomys dilectus*** (de Winton, 1897). English: Mesic Four-striped Grass Mouse. Swahili: Panya. Chinese: 纹鼠. Included within *Rhabdomys striatus* by Happold D (2013w) but now considered as a separate species (Monadjem et al., 2015). Recorded from Zimbabwe, Malawi, Zambia, Tanzania, Angola, Kenya and E Uganda (Monadjem et al., 2015). Grassy and shrubby habitats mostly in highland areas. In Kenya, recorded widely from the highlands in the SW.

**Genus** ***Thallomys*** Thomas, 1920. Acacia Rats

128. ***Thallomys loringi*** (Heller, 1909). English: Loring’s Acacia Rat. Swahili: Panya. Chinese: 洛林青毛鼠. Recorded from W, C and SW Kenya and N Tanzania. Arboreal in *Acacia* trees, shrubby woodlands and brushy thickets in savanna habitats (Carleton, 2013). In Kenya, recorded from scattered localities in the Rift Valley.
129. ***Thallomys paedulcus*** (Sundevall, 1846). English: Sundevall’s Acacia Rat. Swahili: Panya. Chinese: 松德瓦尔青毛鼠. Recorded from S Ethiopia and S Somalia through eastern and Central Africa. Savanna habitats, especially *Acacia* woodlands (Perrin, 2013b). In Kenya, widely distributed in the S, E and N, but its relationship with *T. loringi* remains unresolved.

**Genus** ***Zelotomys*** Osgood, 1910. Broad-headed Mice

130. ***Zelotomys hildegardeae*** (Thomas, 1902). English: Hildegarde’s Broad-headed Mouse. Swahili: Panya. Chinese: 妒鼠. Recorded from SE Central Africa Republic and South Sudan through East Africa to Zambia, S DR Congo and Angola. Moist grassland savanna, edge of swamps and forests, and grasslands (Nel, 2013). In Kenya, occurs widely in the SW.

**Family ANOMALURIDAE**

**Genus** ***Anomalurus*** Waterhouse, 1842. Anomalures

131. ***Anomalurus derbianus*** (Gray, 1842). English: Lord Derby’s Anomalure. Swahili: unavailable. Chinese: 鳞尾松鼠. Recorded widely in West, Central and East Africa. Rainforests, secondary forests and riverine forests, cultivations with large forest trees, and savannas with relict forests. In Kenya, restricted to highland forests in the W (Ray, 2013a).

**Family PEDETIDAE**

**Genus** ***Pedetes*** Illiger, 1811. Springhares

132. ***Pedetes surdaster*** (Thomas, 1902). English: East African Springhare. Swahili: Kamendengere. Chinese: 东非跳兔. Recorded in SC Kenya and C Tanzania. Semi-arid grassland and open habitats (Butynski & Kalina, 2013). In Kenya, recorded from S of Nairobi on grassland plateaux, including Amboseli NP and Masai Mara NR.

**Family BATHYERGIDAE**

**Genus** ***Heliophobius*** Peters, 1846. Silvery Mole-rats

133. ***Heliophobius argenteocinereus*** Peters, 1846. English: Silvery Mole-rat. Swahili: Fuko. Chinese: 霜鼠. Recorded from S Kenya, SE DR Congo, N Zambia, Malawi and N and C Mozambique. *Combretum-Brachystegia* woodlands, rocky hillsides and agricultural fields. In Kenya, recorded from C and S regions (Jarvis, 2013b).

**Family HETEROCEPHALIDAE**

The single genus and species in this family was previously placed in the family Bathyergidae (Jarvis, 2013c) but is now placed in the newly recognized family Heterocephalidae (Patterson & Upham, 2014).

**Genus** ***Heterocephalus*** Ruppell, 1842. Naked Mole-rats

134. ***Heterocephalus glaber*** Rüppell, 1842. English: Naked Mole-rat. Swahili: Fuko. Chinese: 裸鼢鼠. Recorded from Somalia, E Ethiopia and N and SE Kenya. Semi-deserts and arid habitats with hard soil. In Kenya, recorded widely from the NE (Jarvis, 2013c).

**Family HYSTRICIDAE**

**Genus** ***Atherurus*** F. Cuvier, 1829. Brush-tailed Porcupines

135. ***Atherurus africanus*** Gray, 1842. English: African Brush-tailed Porcupine. Swahili: Njiko. Chinese: 非洲帚尾豪猪. Recorded widely from Senegal to Uganda and Kenya, including Cameroon, Gabon and C DR Congo. Rainforests, secondary forests, gallery forests and relict rainforests (Happold D, 2013x). In Kenya, recorded in the W.

**Genus** ***Hystrix*** Linnaeus, 1758. Crested Porcupines

136. ***Hystrix africaeaustralis*** Peters, 1852. English: Cape Crested Porcupine. Swahili: Nungunungu Kusi. Chinese: 南非豪猪. Recorded widely from S DR Congo, Uganda and Kenya throughout the southern part of Africa. Wooded savannas, semi-arid habitats, forests and farmlands (Happold D, 2013y). In Kenya, recorded only from the SW.
137. ***Hystrix cristata*** Linnaeus, 1758. English: North African Crested Porcupine. Swahili: Nungunungu Kishugi. Chinese: 非洲冕豪猪. Recorded widely in NE Africa (Morocco, N Algeria) and from Senegal through N Nigeria, Central African Republic and NE DR Congo to Kenya and Tanzania, with isolated populations in Eritrea and Ethiopia. Semi-deserts, woodland and grassland savannas, rocky hillsides and caves (Happold D, 2013z). In Kenya, widespread.

**Family THRYONOMYIDAE**

**Genus** ***Thryonomys*** Fitzinger, 1867. Cane Rats

138. ***Thryonomys gregorianus*** (Thomas, 1894). English: Lesser Cane Rat. Swahili: Ndezi. Chinese: 草原蔗鼠. Recorded from South Sudan, Uganda and Kenya southwards to Zambia, Malawi and Zimbabwe, with isolated populations in S Chad, Ethiopia, S DR Congo and perhaps Mozambique. Grasslands and rocky habitats in savannas (Happold D, 2013za). In Kenya, recorded widely in the S and W.
139. ***Thryonomys swinderianus*** (Temminck, 1827). English: Greater Cane Rat. Swahili: Ndezi. Chinese: 南撒哈拉蔗鼠. Recorded from Senegal across West Africa to the Central African Republic, Uganda and Kenya, and south to Zambia, Botswana, Zimbabwe and South Africa. Swamps, reedbeds, long grass where damp, sugar cane plantations, and agricultural fields (Happold D, 2013zb). In Kenya, mainly in the W and S, where it is sympatric with *T. gregorianus*, but typically occurs in wetter habitats than the latter species.

**Family MYOCASTORIDAE**

**Genus** ***Myocastor*** Kerr, 1792. Coypu

140. ***Myocastor coypus*** (Molina, 1782). English: Coypu. Swahili: unavailable. Chinese: 河狸鼠. Exotic species in Africa. Introduced into aquatic habitats in southern and eastern Africa. Swamps, rivers, farm ponds and dams. In Kenya, recorded in C regions (e.g., Laikipia, Nanyuki, Aberdare Ranges, Lake Naivasha) (Happold D, 2013zc).

**ORDER LAGOMORPHA (Hares and Rock-hares–three species)**

**Family LEPORIDAE**

**Genus** ***Lepus*** Linnaeus, 1758. Hares and rock-hares

141. ***Lepus capensis*** Linnaeus, 1758. English: Cape Hare; Swahili: Sungura. Chinese: 草兔. Recorded throughout most of the continent, except in desert (and other arid areas) and rainforest; not present from Angola westwards to Mozambique. Grasslands and other open habitats (Happold D, 2013zd). In Kenya, recorded in most of the country, except the NE.
142. ***Lepus victoriae*** Thomas, 1893. English: African Savanna Hare; Swahili: Sungura. Chinese: 海角兔. Recorded from Mauritania through western Africa to Sudan, then southwards through Uganda and Kenya to Angola, Zambia, Zimbabwe, and parts of Botswana and South Africa. Scrubland, bushland and grassland habitats, preferring less open areas to *L. capensis* (Happold D, 2013ze). In Kenya, recorded W of the Rift Valley, with an isolated population on Mt. Kenya (Flux & Flux, 1983).

**Genus** ***Pronolagus*** Lyon, 1904. Rock-Hares

143. ***Pronolagus rupestris*** (A. Smith, 1834). English: Smith’s Red Rock-hare; Swahili: Sungura ya Mawe. Chinese: 红兔. Recorded in two disjunct areas: (1) Kenya, Tanzania, NE Zambia and Malawi in a narrow band and bordering the Rift Valley; (2) NW South Africa. Rocky hillsides with boulders and rocky crevices (Happold D, 2013zf). In Kenya, recorded in the SW, including the Ngong Hills.

**ORDER ERINACEOMORPHA (Hedgehogs–one species)**

**Family ERINACEIDAE**

**Genus** ***Atelerix*** Pomel, 1848. Hedgehogs

144. ***Atelerix albiventris*** (Wagner, 1841). English: White-bellied Hedgehog; Swahili: Kalunguyeye/Nungunungu. Chinese: 白腹刺猬. Recorded from Senegal eastwards across West Africa to Sudan, Somalia and lower elevations of Ethiopia, and then southwards through Kenya, Uganda, Tanzania to Malawi and Zambia. Savanna and semi-arid habitats, including fields and suburban gardens. In Kenya, recorded throughout most of the country (Happold D, 2013zg).

**ORDER SORICOMORPHA (Shrews–36 species)**

The Swahili name for the cryptic, unobtrusive and hard-to-see shrew species of this order is ‘Kirukanjia/Njule’ 

**Family SORICIDAE**

**Genus** ***Crocidura*** Wagler, 1832. White-toothed Shrews

145. ***Crocidura allex*** Osgood, 1910. English: East African Highland Shrew. Chinese: 肯尼亚麝鼩. Recorded from Kenya and N Tanzania. In alpine grasslands and swamp habitats. In Kenya, recorded from C regions (Mau Forest, Aberdare Range, Mt. Kenya) (Hutterer, 2013b).
146. ***Crocidura bottegi*** Thomas, 1898. English: Bottego’s Shrew. Chinese: 博氏麝鼩. Recorded from Kenya and Ethiopia. In *Acacia-Commiphora* bushlands and arid habitats. In Kenya, recorded from the N (Marsabit) (Hutterer, 2013c).
147. ***Crocidura elgonius*** Osgood, 1910. English. Elgon Shrew. Chinese: 埃尔贡麝鼩. Recorded from Kenya and Tanzania. In highland and montane habitats. In Kenya, recorded in C (Muguga Nairobi) and W regions (Cherangani, Mt. Elgon, Nakuru, Eldoret, Kakamega Forest) (Stanley, 2013a).
148. ***Crocidura fischeri*** Pagenstecher, 1885. English: Fischer’s Shrew. Chinese: 费氏麝鼩. Recorded from Kenya and N Tanzania. In grasslands of *Acacia* savanna woodlands, with scattered dominant *Acacia tortilis* trees. In Kenya, recorded from S regions (Nguruman, N of Lake Natron) (Hutterer, 1986; Oguge, 2013a).
149. ***Crocidura fulvastra*** (Sundevall, 1843). English: Savanna Shrew. Chinese: 金色麝鼩. Recorded from Mali, N Nigeria, S and C Sudan, Ethiopia and N Kenya. In drier savanna and arid habitats. In Kenya, recorded in the N (around Lake Turkana) (Churchfield & Jenkins, 2013a).
150. ***Crocidura fumosa*** Thomas, 1904. English. Smoky White-toothed Shrew. Chinese: 烟色麝鼩. Endemic to Kenya and restricted to moist montane forest habitats of the E slopes Mt. Kenya and Aberdare Ranges (Churchfield & Jenkins, 2013b). Specimens from Mt. Kenya were studied genetically by Stanley et al. (2015).
151. ***Crocidura fuscomurina*** (Heuglin, 1865). English: Bicoloured Musk Shrew. Chinese: 纺锤麝鼩. Recorded from many countries in West, East, East-Central and southern Africa. In woodland savannas and semi-arid habitats. In Kenya, widely distributed (Dippenaar & Baxter, 2013).
152. ***Crocidura hildegardeae*** Thomas, 1904. English: Hildegarde’s Shrew. Chinese: 尼日利亚麝鼩. Recorded from SE Cameroon and Congo to Kenya and Tanzania. In dry forests and wetter forests of montane and highland areas. In Kenya, recorded W of the Rift Valley from the NW-SE (Stanley, 2013b).
153. ***Crocidura jacksoni*** Thomas, 1904. English: Jackson’s Shrew. Chinese: 杰克逊麝鼩. Recorded from Uganda, Kenya, N Tanzania and E DR Congo. In moist forests, wet bushlands and cultivated areas. In Kenya, widely found W of the Rift Valley (Oguge, 2013b).
154. ***Crocidura littoralis*** Heller, 1910. English: Naked-tail Shrew. Chinese: 滨海麝鼩. Recorded from SW Central African Republic, Cameroon, Congo, DR Congo, Uganda and Kenya. In closed-canopy rainforests. Restricted to W Kenya (Ray & Hutterer, 2013).
155. ***Crocidura luna*** Dollman, 1910. English: Moonshine Shrew. Chinese: 新月麝鼩. With more than one species, this complex needs taxonomic revision (Castiglia et al., 2009). Recorded from NE DR Congo, Uganda, Kenya, most of Tanzania, Zambia, SE DR Congo, WC Mozambique, Malawi and Mozambique. In moist, cool areas, typically on the fringes of montane forests with dense cover and in matted grass along streams. In Kenya, recorded from the S and W (Baxter & Dippenaar, 2013a).
156. ***Crocidura macarthuri*** St. Leger, 1934. English: MacArthur’s Shrew. Chinese: 麦氏麝鼩. Recorded from S Kenya (Nguruman) and C Somalia. In wooded grasslands with widely scattered *Acacia* trees, e.g., *Acacia tortilis*, interspersed with *Themeda*, *Hyparrhenia* and *Cenchrus* grasses (S Kenya) (Oguge, 2013c) and Meru National Park (Hutterer, unpublished data).
157. ***Crocidura macowi*** Dollman, 1915. English: Nyiro Shrew. Chinese: 麦考麝鼩. Endemic to Kenya and recorded from Mt. Nyiro and S of Lake Turkana in tropical forests (Churchfield & Jenkins, 2013c).
158. ***Crocidura monax*** Thomas, 1910. English: Kilimanjaro Shrew. Chinese: 僧麝鼩. Recorded from N Mt. Pare and Mt. Kilimanjaro in Tanzania, and also possibly present on the Kenyan side of Mt. Kilimanjaro (Stanley et al., 2015). In moist montane forest habitats.
159. ***Crocidura montis*** Thomas, 1906. English: Montane White-toothed Shrew. Chinese: 山林麝鼩. This species is possibly restricted to Mt. Rwenzori, DR Congo (Stanley et al., 2015). Other populations in East Africa may represent different species, which require revision. In montane grasslands. In Kenya, recorded under this name from C and W regions (Hutterer, 2013d).
160. ***Crocidura nanilla*** Thomas, 1909. English: Savanna Dwarf Shrew. Chinese: 西非麝鼩. Recorded from Mauritania to E Africa (Happold D, 2013zh). Species may be composite; Thorn & Kerbis Peterhans (2009) restricted the type locality of *C. nanilla* to the “Rift Valley of central Kenya, probably near Kinangop, approximately S0°45′ E36°30′.
161. ***Crocidura nigrofusca*** Matschie, 1895. English: African Black Shrew. Chinese: 非洲黑麝鼩. Recorded from East Africa as well as S Ethiopia, southern Sudan, Zambia, Angola, C and S DR Congo, Zambia and Malawi. In damp habitats near water courses. In Kenya, widely distributed (Hutterer, 2013e; Oguge et al., 2004).
162. ***Crocidura olivieri*** (Lesson, 1827). English: African Giant Shrew. Chinese: 非洲大麝鼩. Very widespread, but polytypic African shrew recorded from western to southern Africa, including Egypt. In a wide variety of habitats. In Kenya, widespread (Churchfield & Hutterer, 2013; Jacquet et al., 2015).
163. ***Crocidura parvipes*** Osgood, 1910. English: Small-footed Shrew. Chinese: 小足麝鼩. Recorded from East, Central and southern Africa. In dry savanna, mixed forest and gallery forest habitats. In Kenya, widely distributed W of the Rift Valley (Hutterer, 2013f).
164. ***Crocidura raineyi*** Heller, 1912. English: Rainey’s Shrew. Chinese: 雷氏麝鼩. Endemic to Kenya and recorded from Mt. Gargues and Matthews Range. In montane forests and along creeks (Hutterer, 2013g).
165. ***Crocidura selina*** Dollman, 1915. English: Uganda Lowland Shrew. Chinese: 甘蓝麝鼩. Recorded from Uganda and SE (Chyulu Hills) Kenya. In lowland evergreen forest habitats (Hutterer, 2013h). Specimens from Chyulu Hills are similar but not yet fully studied (Oguge et al., 2004).
166. ***Crocidura turba*** Dollman, 1910. English: Turbo Shrew. Chinese: 安哥拉麝鼩. Recorded from NW Cameroon to Kenya southwards to Zambia and Angola. In dry forest, montane forests, riverine habitats, bushlands and grasslands. In Kenya, recorded from W of the Rift Valley (Oguge, 2013d).
167. ***Crocidura ultima*** Dollman, 1915. English: Ultimate Shrew. Chinese: 罕麝鼩. Endemic to Kenya and recorded from the Jombeni Range, near Nyeri. In montane tropical moist forest (Churchfield & Jenkins, 2013d).
168. ***Crocidura viaria*** (I. Geoffroy, 1834). English: Savanna Path Shrew. Chinese: 路麝鼩. Recorded from S Morocco to Senegal and eastwards to Sudan, W Ethiopia and Kenya. In cultivated fields, sand dunes and dense shrubby vegetation. In Kenya, widely distributed (Hutterer, 2013i).
169. ***Crocidura voi*** Osgood, 1910. English: Voi Shrew. Chinese: 沃伊麝鼩. Recorded from Kenya, Somalia, Ethiopia and Sudan, including a single record in Nigeria and Mali. In very dry savannas and coastal forests. In Kenya, widely distributed (Happold D, 2013zi).
170. ***Crocidura xantippe*** Osgood, 1910. English: Xanthippe’s Shrew. Chinese: 尼鲁麝鼩. Recorded from SE Kenya and Tanzania. In a wide variety of habitats (Stanley, 2013c).
171. ***Crocidura yankariensis*** Hutterer & Jenkins, 1980. English: Yankari Shrew. Chinese: 博契麝鼩. Recorded from Nigeria, Cameroon, Sudan, Ethiopia, Kenya and Somalia. In dry savannas. In Kenya, recorded from the NW (W Turkana, Lotikipi area). (Hutterer, 2013j).
172. ***Crocidura zaphiri*** Dollman, 1915. English: Zaphir’s Shrew. Chinese: 扎氏麝鼩. Taxonomical status unknown. Recorded from S Ethiopia and W Kenya (Kaimosi and Kisumu). In tropical forests (Churchfield & Jenkins, 2013e).

**Genus** ***Suncus*** Ehrenberg, 1832. Pygmy and House Shrews

173. ***Suncus aequatorius*** (Heller, 1912). English: Taita Dwarf Shrew. Chinese: 泰塔臭鼩. Recorded from SE Kenya (Taita Hills-Summit of Mt. Sagalla and Chawia Forest) and N Tanzania. In disturbed remnant forest in highland areas (Oguge & Hutterer, 2013).
174. ***Suncus infinitesimus*** (Heller, 1912). English: Least Dwarf Shrew. Chinese: 肯尼亚臭鼩. Recorded from South Africa, Kenya, Central African Republic and Cameroon. In primary montane forests and grasslands, savannas and mixed bushveld. In Kenya, recorded from C regions (Rumuruti and Rongai) (Baxter & Dippenaar, 2013b).
175. ***Suncus megalura*** (Jentink, 1888). English: Climbing Shrew. Chinese: 大尾臭鼩. Allocation to genus *Suncus* provisional (Hutterer, 2005). Recorded from West, Central and East Africa and southwards into E Zimbabwe, C Mozambique and Angola. In a wide variety of habitat but mostly associated with moist savanna. In Kenya, restricted to the S and W (Baxter & Dippenaar, 2013c).
176. ***Suncus murinus*** (Linnaeus, 1766). English: Asian House Shrew. Chinese: 臭鼩. Introduced exotic species in disturbed and natural habitats along the coast from Egypt to Tanzania, as well as around Lake Victoria (Duplantier, 2013). In Kenya, recorded along the coast and Lake Victoria.

**Genus** ***Surdisorex*** Thomas, 1906. Mole-shrews

177. ***Surdisorex norae*** (Thomas, 1906). English: Aberdare Mole-shrew. Chinese: 肯尼亚聋鼠鼩鼱. Endemic to Kenya and recorded from C regions (Aberdare Ranges). In swamps in moorlands (Happold D, 2013zj).
178. ***Surdisorex polulus*** Hollister, 1916. English: Mount Kenya Mole-shrew. Chinese: 小聋鼠鼩鼱. Endemic to Kenya and recorded from C regions (W of Mt. Kenya) in *Podocarpus*-bamboo and swamps near forests (Happold D, 2013zk).
179. ***Surdisorex schlitteri*** Kerbis Peterhans, Stanley, Hutterer, Demos & Agwanda, 2009. English: Schlitters’s Mole-shrew. Chinese: 施氏鼠鼩鼱. Recorded from Mt. Elgon sides of Kenya and Uganda. In montane habitats (Kerbis et al., 2009).

**Genus** ***Sylvisorex*** Thomas, 1904. Forest Shrew

180. ***Sylvisorex mundus*** Osgood 1910. English: Osgood’s Forest Shrew. Chinese: 奥氏林鼩鼱. Demos et al. (2014, 2015) justified use of this name for Kenyan populations, formerly treated as *S. granti*. Recorded from East Africa. In swamps, montane forests and damp bushy vegetation above 1 500 m. In Kenya, recorded from W (Mt. Elgon, Cherangani Hills) and C regions (Mt. Kenya, Aberdare Ranges) (Dieterlen, 2013t as *S. granti*).

**ORDER CHIROPTERA (Bats–104 species)**

The Kenyan bat fauna includes members of all 11 families of bats known from Africa (Patterson & Webala, 2012). The Swahili name for bat is “Popo”, and currently no other name exists for different species of bats found in Kenya. 

**Family PTEROPODIDAE**

**Genus** ***Eidolon*** Rafinesque, 1815. Straw-coloured Fruit Bats

181. ***Eidolon helvum*** (Kerr, 1792). English: African Straw-coloured Fruit Bat. Chinese: 黄毛果蝠. Recorded widely in sub-Saharan Africa. In all forests and woodland savannas with trees producing enough fruit. In Kenya, recorded from W and C regions and in wetter areas along the Kenya-Tanzania border from the SW to the coastal strip as far as Pate Island, Lamu (Thomas & Henry, 2013a).

**Genus** ***Epomophorus*** Bennett, 1835. Epauletted Fruit Bats

182. ***Epomophorus labiatus*** (Temminck, 1837). English: Little Epauletted Fruit Bat. Chinese: 小颈囊果蝠. Recorded disjunctly from NE Nigeria, S Chad and S Congo, and in some areas from C Sudan to Eritrea, Ethiopia and Djibouti and southwards to N Zambia and S Malawi. Found in a wide variety of woodland savannas (Happold M, 2013a). In Kenya, recorded from the W and SE half of the coastal strip.
183. ***Epomophorus minimus*** Claessen & De Vree, 1991. English: Least Epauletted Fruit Bat. Chinese: 侏颈囊果蝠. Recorded disjunctly in eastern Africa, including S Sudan and Ethiopia. Found in a wide variety of habitats, usually near rivers or highlands in the E (Happold M, 2013b). In Kenya, recorded disjunctly from N, C and E regions (Claessen & De Vree, 1991).
184. ***Epomophorus wahlbergi*** (Sundevall, 1846). English: Wahlberg’s Epauletted Fruit Bat. Chinese: 韦氏颈囊果蝠. Recorded widely in Central, eastern and southern Africa; in various woodland and forests habitats (Happold M, 2013c). In Kenya, widely distributed, mostly W of the Rift Valley and along the coastal strip.

**Genus** ***Hypsignathus*** H. Allen, 1861. Hammer-headed Fruit Bat

185. ***Hypsignathus monstrosus*** H. Allen, 1861. English: Hammer-headed Fruit Bat. Chinese: 锤头果蝠. Recorded from West Africa to Uganda and W Kenya (with outliers in W Ethiopia), and southwards to NW Angola and DR Congo. Found mostly in lowland rainforests, swamp forests and surrounding mosaics of these forests and secondary grasslands, but also in palm forests, riverine forests and mangroves (Happold M, 2013d). In Kenya, recorded only in the W and near Kakamega Forest (Aggundey & Schlitter, 1984).

**Genus** ***Micropteropus*** Matschie, 1899. Lesser Epauletted Fruit Bats

186. ***Micropteropus pusillus*** (Peters, 1868). English: Peters’s Lesser Epauletted Fruit Bat. Chinese: 非洲小狐蝠. Recorded from Senegal to W Ethiopia and southwards (disjunctly) to Angola and S DR Congo; mostly from low-elevation savanna-forest ecotones (uncommon in closed rainforest) (Thomas & Henry, 2013b). In Kenya, recorded only in the W (Aggundey & Schlitter, 1984; Patterson & Webala, 2012).

**Genus** ***Myonycteris*** Matschie, 1899. Collared Fruit Bats

187. ***Myonycteris angolensis*** (Bocage, 1898). English: Angolan Collared Fruit Bat. Chinese: 安哥拉领果蝠. This species was formerly referred to as *Lissonycteris angolensis*, but a recent molecular review of the tribe Myonycterini placed *Lissonycteris* within the genus *Myonycteris* (Nesi et al., 2013). Disjunct records from Senegal to the Ethiopian highlands, and southwards to S20°. Found in rainforests, montane forests, forest-savanna mosaics and, to a lesser extent, woodland savanna habitats (Happold M, 2013e as *Lissonycteris angolensis*). In Kenya, recorded from the W, C and SE.
188. ***Myonycteris relicta*** Bergmans, 1980. English: Bergmans’ Collared Fruit Bat. Chinese: 孤领果蝠. Disjunct records from SE Kenya, coastal and C Tanzania and E Zimbabwe. Found in forests of the East African coastal mosaics and inland lowland forests (but not in East African savannas) (Taylor, 2013d). In Kenya, recorded S of Mombasa City in the SE (Shimba Hills, Lukore area-Makanda River).

**Genus** ***Rousettus*** Gray, 1821. Rousettes

189. ***Rousettus aegyptiacus*** (É. Geoffroy, 1810). English: Egyptian Rousette. Chinese: 北非果蝠. Disjunct records from the Nile Valley in Egypt and from most (but not all) countries in sub-Saharan Africa. Found in a wide variety of habitats where caves and fruiting trees are present (Happold M, 2013f). In Kenya, widely distributed in the western half of the country, C and extending to the southern half of the coastal strip.

**Genus** ***Stenonycteris*** Andersen, 1912. Long-haired Rousette

The genus *Stenonycteris* has recently been validated as distinct from the genus *Rousettus* (Nesi et al., 2013).

190. ***Stenonycteris lanosus*** (Thomas, 1906). English: Long-haired Rousette. Chinese: 狭齿果蝠. Recorded from eastern Africa from SW Ethiopia to S Sudan, E DC Congo, W Kenya, E Tanzania and N Malawi. Found mostly in or near afro-montane vegetation, but also recorded in mosaics of evergreen bushland and secondary *Acacia* woodland, and occasionally in drier lowland rainforest, miombo woodland and various bushland and thicket habitats (Happold M, 2013g as *Rousettus lanosus*). In Kenya, recorded from several C and W localities.

**Family RHINOLOPHIDAE**

**Genus** ***Rhinolophus*** Lacépède, 1799. Horseshoe Bats

191. ***Rhinolophus clivosus*** Cretzschmar, 1828. English: Geoffroy’s Horseshoe Bat. Chinese: 佐氏菊头蝠. Disjunct records from northern, central, eastern and southern Africa, including the Horn of Africa. Found in a wide variety of habitats (Bernard & Happold M, 2013a). In Kenya, recorded widely in the NW and mid-W to C (including in caves in Naivasha and Mt Elgon National Park) and SE regions (Taita Hills) (López-Baucells et al., 2016).
192. ***Rhinolophus deckenii*** Peters, 1868. English: Decken’s Horseshoe Bat. Chinese: 德氏菊头蝠. Recorded only in East Africa, including Mafia, Zanzibar and Pemba islands and Mozambique. Found mostly in coastal forests (Happold M, 2013h; Monadjem et al., 2010). In Kenya, recorded only in coastal forests in the East African coastal forest mosaics.
193. ***Rhinolophus eloquens*** K. Andersen, 1905. English: Eloquent Horseshoe Bat. Chinese: 乌干达菊头蝠. Recorded only from eastern Africa, including E DR Congo, Rwanda, South Sudan and Somalia. Semi-arid savannas (including *Acacia-Commiphora* deciduous bushland and thickets, and mosaics of evergreen bushland and secondary *Acacia* wooded grassland), mesic woodland savannas and montane forests (Cotterill, 2013a). In Kenya, widely distributed, mostly W of the Rift Valley.
194. ***Rhinolophus fumigatus*** Rüppell, 1842. English: Rüppell’s Horseshoe Bat. Chinese: 达马拉菊头蝠. Highly disjunct records from Senegal to Cameroon in West Africa, and also disjunctions from NE Gabon and N Congo southwards to Angola and Namibia, and from Eritrea, E Sudan, Ethiopia and NE DR Congo southwards to NE South Africa. Found in woodland savannas (Cotterill & Happold M, 2013a). In Kenya, widely distributed in N-C regions (including Laikipia, Meru and Marsabit) and extending to the southern half of the coastal strip, mostly in *Acacia-Commiphora* deciduous bushland and thickets.
195. ***Rhinolophus hildebrandtii*** Peters, 1878. English: Hildebrandt’s Horseshoe Bat. Chinese: 希氏菊头蝠. Currently considered to occur only in S Ethiopia and East Africa; in semi-arid and mesic woodland savannas and riverine forests (Taylor et al., 2012; Cotterill & Happold M, 2013b). Previous records from further S are now considered to represent other species (Taylor et al., 2012). In Kenya, widely recorded W of the Rift Valley and extending to the SE (including Chyulu Hills).
196. ***Rhinolophus landeri*** Martin, 1837 (publ. 1838). English: Lander’s Horseshoe Bat. Chinese: 兰德菊头蝠. Widespread but disjunct records in sub-Saharan Africa from Senegal to Ethiopia and southwards to NE South Africa. Found in a very wide variety of habitats (Happold M, 2013i). A recent study showed that savanna populations in South and East Africa represent a distinct species of *R. lobatus* (Taylor et al., 2018), which is likely widely distributed in Kenya.
197. ***Rhinolophus simulator*** K. Andersen, 1904. English: Bushveld Horseshoe Bat. Chinese: 布什维尔德菊头蝠. Highly disjunct records in West Africa (Guinea to W Cameroon) and also disjuncts from C Ethiopia and S Sudan to NE South Africa. Found in various habitats, including rainforests, montane forests, wetter woodland savannas, coastal mosaics and valley bushveld, but probably only near caves and/or abandoned mines (Cotterill & Happold M, 2013c). In Kenya, recorded from the W and SE.

**Family HIPPOSIDERIDAE**

Species traditionally treated as within the genus *Hipposideros* have been recently allocated to three different genera, namely *Hipposideros*, *Doryrhina* and *Macronycteris* (Foley et al., 2017).

**Genus** ***Doryrhina*** Peters, 1871. Leaf-nosed Bats

Foley et al. (2017) placed *Hipposideros cyclops* in *Doryrhina*, but their study did not include *H. camerunensis*. Because *cyclops* and *camerunensis* are traditionally considered to be very closely related, we provisionally placed *camerunensis* in *Doryrhina*; however, this needs confirmation.

198. ***Doryrhina camerunensis*** (Eisentraut, 1956). English: Cameroon Leaf-nosed Bat. Chinese; 喀麦隆蹄蝠. Originally described as *Hipposideros camerunensis* but see Genus *Doryrhina* above. Disjunct records from S Cameroon, E DR Congo and W Kenya. Found in montane and lowland rainforests (Happold M, 2013j as *Hipposideros camerunensis*). In Kenya, recorded only from North Nandi and Kakamega forests in the W, in degraded montane forest and intermediate evergreen forest, respectively.
199. ***Doryrhina cyclops*** (Temminck, 1853). English: Cyclops Leaf-nosed Bat. Chinese: 大眼蹄蝠. Disjunct records from Senegal to coastal Kenya and Tanzania. Found mostly in lowland rainforests, but also in coastal, montane, swamp and mangrove forests (Fahr, 2013a as *Hipposideros cyclops*). In Kenya, recorded only from small areas in the SW and SE.

**Genus** ***Hipposideros*** Gray, 1831. Old World Leaf-nosed Bats

200. ***Hipposideros caffer*** (Sundevall, 1846). English: Sundevall’s Leaf-nosed Bat. Chinese: 松氏蹄蝠. Widespread in many African countries. Found in woodland savannas (Bernard & Happold M, 2013b). In Kenya, *H. caffer* is widely distributed from W of the Rift Valley to the C and E along the coastal strip. However, taxonomic revision is required as *H. caffe*r is considered to be a species complex (Kock et al., 2008; Vallo et al., 2008). Three subspecies were listed by Simmons (2005), including: *H. c. angolensis* Seabra, 1898; *H. c. nanus* J.A. Allen, 1917; and *H. c. tephrus* Cabrera, 1906. However, a study by Vallo et al. (2008) revealed two distinct clades, *H. c. caffer* and *H. c. tephrus*, respectively, inhabiting southern Africa and the Maghreb, West Africa and Arabian Peninsula. Whether East African populations belong to either of the two clades or to a distinct one requires investigation.
201. ***Hipposideros megalotis*** (Heuglin, 1862). English: Large-eared Leaf-nosed Bat. Chinese: 串耳蹄蝠. Recorded from Eritrea, Ethiopia, Djibouti and Kenya. Found in afro-montane vegetation and various wooded grasslands, bush lands and thickets, and semi-desert grasslands (Happold M, 2013k). In Kenya, recorded in W and C regions.
202. ***Hipposideros ruber*** (Noack, 1893). English: Noack’s Leaf-nosed Bat. Chinese: 诺氏蹄蝠. Vallo et al. (2008) recognized several species lineages within the *caffer-ruber* complex throughout Africa, a view supported by Monadjem et al. (2013b). Molecular data are, therefore, required to resolve the taxonomy of the group. In fact, according to Vallo et al. (2008), *H. ruber* is only found in East Africa. In Kenya, the species is only recorded from the W and SE (Happold M, 2013l).

**Genus** ***Macronycteris*** Gray, 1866. Leaf-nosed Bats

203. ***Macronycteris gigas*** (Wagner, 1845). English: Giant Leaf-nosed Bat. Chinese: 巨蹄蝠. Recorded disjunctly from West, Central and East Africa. Found in lowland tropical rainforests, East African coastal forests and wetter woodland savannas. In Kenya, recorded only from the SE in the coastal strip (Happold M, 2013m as *Hipposideros gigas*).
204. ***Macronycteris vittata*** (Peters, 1852). English: Striped Leaf-nosed Bat. Chinese: 大白纹蹄蝠. Highly disjunct records from N Nigeria and N Cameroon, from the eastern side of Africa (from Ethiopia and Somalia to NE South Africa), and from SW Angola. Found in a wide variety of habitats. In Kenya, recorded only in the SE, in coastal forests and adjacent bushland (Happold M, 2013n as *Hipposideros vittatus*).

**Family RHINONYCTERIDAE**

Following Foley et al. (2015, 2017), we recognize the family Rhinonycteridae as distinct from the family Hipposideridae, to which we allocate the genera *Cloeotis* and *Triaenops*.

**Genus** ***Cloeotis*** Blyth, 1848. Percival’s Trident Bat

205. ***Cloeotis percivali*** Thomas, 1901. English: Percival’s Trident Bat. Chinese: 珀氏三叉蝠. Disjunct records in Kenya, Mafia Island, Tanzania, and also from SE DR Congo to Swaziland and NE South Africa. Mostly found in undifferentiated woodlands, wetter and drier miombo woodlands and mopane woodlands (Jacobs, 2013). In Kenya, only recorded in coastal forests N and S of Mombasa.

**Genus** ***Triaenops*** Dobson, 1871. Trident Bat

206. ***Triaenops afer*** Dobson, 1871. English: African Trident Bat. Chinese: 非洲三叉蝠. Highly disjunct records from S Central African Republic Africa to NW Angola, and from Ethiopia, NE DR Congo and Somalia southwards to E Zimbabwe and S Mozambique. Found in various habitats, including coastal forests, riverine forests and farmlands with patches of miombo woodland and/or remnant rainforest (Happold M, 2013o). In Kenya, recorded widely from the NW to SE, as well as coastal forests and drier habitats, including *Acacia-Commiphora* deciduous bushland and thickets.

**Family MEGADERMATIDAE**

**Genus** ***Cardioderma*** Peters, 1873. Heart-nosed Bat

207. ***Cardioderma cor*** (Peters, 1872). English: Heart-nosed Bat. Chinese: 非洲假吸血蝠. Recorded widely with disjunctions from Sudan, Eritrea, Djibouti, Ethiopia and Somalia to Uganda, Kenya and NE Tanzania, including Zanzibar Island. Found in dry and moist habitats in *Acacia-Commiphora* deciduous bushland and thickets, semi-desert grasslands and coastal forests (Happold M, 2013p). In Kenya, very widespread from the NW to SE.

**Genus** ***Lavia*** Gray, 1838. Yellow-winged Bat

208. ***Lavia frons*** (É. Geoffroy, 1810). English: Yellow-winged Bat. Chinese: 黄翼蝠. Widespread but disjunct records from Senegal to Eritrea and W Somalia (although not most of Ethiopia) and southwards to Gabon, DR Congo, C Zambia, N Malawi and Tanzania. Mostly found in woodland savannas with abundant acacias, but also various other habitats (excluding closed rainforest) (Happold M, 2013q). In Kenya, widely distributed W of the Rift Valley and along the coastal strip.

**Family RHINOPOMATIDAE**

**Genus** ***Rhinopoma*** É. Geoffroy, 1818. Mouse-tailed Bats

209. ***Rhinopoma macinnesi*** Hayman, 1937. English: Macinnes’s Mouse-tailed Bat. Chinese: 麦氏鼠尾蝠. Recorded only from S Eritrea, NE Somalia and NW Kenya. Found in semi-desert vegetation, including grasslands, shrublands, *Acacia-Commiphora* deciduous bushland and thickets (Aulagnier, 2013). In Kenya, recorded in a narrow band from areas around Lake Turkana to Lake Baringo.

**Family EMBALLONURIDAE**

**Genus** ***Coleura*** Peters, 1867. Sheath-tailed Bats

210. ***Coleura afra*** (Peters, 1852). English: African Sheath-tailed Bat. Chinese: 非洲鞘尾蝠. Disjunct records from parts of West Africa, and in an area bounded by the Central African Republic, Red Sea coast in Sudan, W Tanzania and SE Kenya (but not everywhere), and also in mid-W Angola and W Mozambique. Found in coastal habitats, woodland savannas, drier bushlands and thicket-scrubs (Happold M, 2013r). In Kenya, moderately widespread from the western border and across C parts to the coastal strip.

**Genus** ***Saccolaimus*** Temminck, 1838. Pouched Bats

211. ***Saccolaimus peli*** (Temminck, 1853). English: Pel’s Pouched Bat. Chinese: 贝尔墓蝠. Disjunct records from parts of West and Central Africa (including DR Congo and Angola), and from Uganda and W Kenya. Found in rainforest zones, mostly in lowland, coastal and swamp forests but also in montane forests, mangroves, forest-savanna mosaics and (rarely) in miombo woodland and *Isoberlinia* woodland (Fahr, 2013b). In Kenya, recorded only in the W (Nandi Forest and Kaimosi).

**Genus** ***Taphozous*** É. Geoffroy, 1818. Tomb Bats

212. ***Taphozous hamiltoni*** Thomas, 1920. English: Hamilton’s Tomb Bat. Chinese: 苏丹墓蝠. Highly disjunct records from S Chad, S Sudan, NE Somalia, Uganda, NW and C Kenya and NW Tanzania. Found in various woodlands, wooded grasslands, deciduous bushland and thickets, and semi-desert grassland and shrubland (Happold M, 2013s). In Kenya, recorded from NW (near Lake Turkana) and C regions (Lake Baringo).
213. ***Taphozous hildegardeae*** Thomas, 1909. English: Hildegarde’s Tomb Bat. Chinese: 肯尼亚墓蝠. Recorded in SE Kenya and NE Tanzania, perhaps including Zanzibar Island. Found near the coast (McWilliam & Happold M, 2013). In Kenya, recorded in the SE near coral caves in remnant coastal forest: two inland records need confirmation (McWilliam & Happold M, 2013).
214. ***Taphozous mauritianus*** É. Geoffroy, 1818. English: Mauritian Tomb Bat. Chinese: 南非墓蝠. Recorded widely throughout most of sub-Saharan Africa (except Ethiopia and Horn of Africa). Found in woodland savannas, large open areas within rainforests, and scattered areas in Sahel savanna (Happold M, 2013t). In Kenya, widespread in the NW, W and S, and along the coastal strip.
215. ***Taphozous nudiventris*** Cretzschmar, 1830. English: Naked-rumped Tomb Bat. Chinese: 裸腹墓蝠. Widely scattered records N of the Equator (but not in all countries) and from N Tanzania. Found in woodland savannas, *Acacia-Commiphora* deciduous bushland and thickets, and more arid semi-desert and desert habitats (Happold M, 2013u). In Kenya, recorded only in the NW.
216. ***Taphozous perforatus*** É. Geoffroy, 1818. English: Egyptian Tomb Bat. Chinese: 埃及墓蝠. Highly disjunct records in parts of West Africa W of NW Nigeria, and in eastern Africa (excluding most of the Horn of Africa) from the Nile Delta to S Zimbabwe. Mostly found in open woodland savannas, moist habitats along the Nile Valley and Okavango Swamp in Botswana, and the East African coastal mosaics (Taylor, 2013e). In Kenya, recorded only in the NW, C and SE along the coastal strip.

**Family NYCTERIDAE**

**Genus** ***Nycteris*** G. Cuvier and É. Geoffroy, 1795. Slit-faced Bats.

217. ***Nycteris arge*** Thomas, 1903. English: Bates’s Slit-faced Bat. Chinese: 淡色凹脸蝠. Widespread but disjunct records from Sierra Leone to South Sudan, SW Kenya and NW Tanzania, and southwards to N Angola and S DR Congo. Mostly found in lowland rainforests, coastal forests and forest-savanna mosaics, but also in montane and swamp forests, in or near relict and riverine forests in the Guinea Savanna, *Acacia-Commiphora* deciduous bushland and thickets, and miombo woodland (Fahr, 2013c). In Kenya, recorded only from the W (Yala River, Kavirondo).
218. ***Nycteris aurita*** (K. Andersen, 1912). English: Andersen’s Slit-faced Bat. Chinese: 安氏凹脸蝠. Disjunct records from Ethiopia, Somalia, Kenya and Tanzania. Found in *Acacia-Commiphora* deciduous bushland and thickets (sometimes near rivers and riverine forests) and coastal forests (Van Cakenberghe & Happold M, 2013a). In Kenya, recorded from the NW and coastal strip.
219. ***Nycteris grandis*** Peters, 1865. English: Large Slit-faced Bat. Chinese: 魁凹脸蝠. Recorded widely from Senegal to NE DR Congo (in rainforest and rainforest mosaics), with apparently separate populations in Kenya and Tanzania (in East African coastal forest mosaics) and some scattered records in Zambia, S Malawi and S Mozambique (in woodlands and riverine forests near large rivers) (Happold M, 2013v). In Kenya, recorded from the SE in coastal forest mosaics.
220. ***Nycteris hispida*** (Schreber, 1775). English: Hairy Slit-faced Bat. Chinese: 粗毛凹脸蝠. Recorded very widely in sub-Saharan Africa (except the Horn of Africa and most of south-western Africa). Found in a wide range of vegetation types (Happold M, 2013w). In Kenya, very widely distributed except in the NE.
221. ***Nycteris macrotis*** Dobson, 1876. English: Large-eared Slit-faced Bat. Chinese: 大耳凹脸蝠. Recorded widely (but with large gaps) in sub-Saharan Africa as far S as NE Angola, NE Botswana, N Zimbabwe and S Mozambique, and also along the River Nile in the Sudan. Found in lowland rainforest, savanna habitats coastal forests and woodlands (Cotterill & Happold M, 2013d). In Kenya, recorded in the NW and widely in the southern half of the country in various habitats, including *Acacia-Commiphora* deciduous bushland and thickets, and coastal forests.
222. ***Nycteris nana*** (K. Andersen, 1912). English: Dwarf Slit-faced Bat. Chinese: 侏凹脸蝠. Highly disjunct records from Côte d’Ivoire and Ghana, Cameroon, parts of Central Africa, and Uganda and Kenya. Found in lowland rainforests, rainforest-savanna mosaics and montane forests, and less often in woodland savannas and coastal forests (Fahr, 2013d). In Kenya, recorded from the W.
223. ***Nycteris thebaica*** É. Geoffroy, 1818. English: Egyptian Slit-faced Bat. Chinese: 非洲凹脸蝠. Recorded widely in many African countries. Found in a wide variety of habitats (although only marginally in rainforest) (Bernard & Happold M, 2013c). In Kenya, recorded widely, except in the NE.

**Family MOLOSSIDAE**

African molossids are represented by at least six genera, including *Tadarida*. Traditionally, *Tadarida* contained several subgenera, including *Chaerephon* and *Mops* (cf. Happold M, 2013x). However, most subsequent phylogenetic work treated them as separate genera, a course adopted here following Lamb et al. (2011), Gregorin & Cirranello (2016) and Naidoo et al. (2016).

**Genus** ***Chaerephon*** Dobson, 1874. Free-tailed Bats

224. ***Chaerephon ansorgei*** (Thomas, 1913). English: Ansorge’s Free-tailed Bat. Chinese: 安氏犬吻蝠. Highly disjunct records in sub-Saharan Africa from NE Ghana to Ethiopia, N Angola, and East Africa to E South Africa. Found mostly in woodland savannas and montane habitats (Cotterill, 2013b as *Tadarida ansorgei*). In Kenya, recorded only W of the Rift Valley.
225. ***Chaerephon bemmeleni*** (Jentink, 1879). English: Gland-tailed Free-tailed Bat. Chinese: 腺尾犬吻蝠. Disjunct records from some parts of West Africa, from one locality in C DR Congo, and from S South Sudan, E DR Congo, S Uganda, SW Kenya and N Tanzania. Found in lowland rainforests and semi-deciduous forests, forest-savanna mosaics, montane grassland, *Isoberlinia* woodland and *Acacia-Commiphora* deciduous bushland (Fahr, 2013e as *Tadarida bemmeleni*). In Kenya, recorded from the SW to SE (but not the coastal strip).
226. ***Chaerephon bivittatus*** (Heuglin, 1861). English: Spotted Free-tailed Bat. Chinese: 斑犬吻蝠. Disjunct records from eastern Africa from Eritrea to Zimbabwe (excluding the Horn of Africa). Mostly found in savanna woodlands and montane habitats (Cotterill, 2013c as *Tadarida bivittata*). In Kenya, recorded from the SW to C and in the SE.
227. ***Chaerephon chapini*** J.A. Allen, 1917. English: Pale Free-tailed Bat. Chinese: 查平犬吻蝠. Recorded disjunctly in several countries in sub-Saharan Africa. Found in woodland savannas and mosaics of rainforest and secondary forests. In Kenya, recorded in the NW (Happold M & Cotterill, 2013 as *Tadarida chapini*).
228. ***Chaerephon major*** (Trouessart, 1897). English: Lappet-eared Free-tailed Bat. Chinese: 垂耳犬吻蝠. The relationship between *major* and other species of *Chaerephon* listed here needs further investigation. Recorded very disjunctly in West Africa (from W Liberia to Nigeria, and perhaps Senegal), from the Nile Valley in Sudan and South Sudan, and from two separate areas in East Africa. Mostly found in woodland and grassland savannas, and riverine habitats along the River Nile and its tributaries (Happold M, 2013y as *Tadarida major*). In Kenya, recorded disjunctly from the area around Lake Victoria and from the SE (coastal strip).
229. ***Chaerephon pumilus*** (Cretzschmar, 1830–1831). English: Little Free-tailed Bat. Chinese: 小犬吻蝠. Recorded from most of West Africa, and from parts of eastern, Central and southern Africa but with many gaps. Mostly found in woodland savannas and forest-savanna mosaics (Happold M, 2013z as *Tadarida pumila*). Probably absent from arid areas, except near rivers. In Kenya, widespread, except in most of the N.
230. ***Chaerephon russatus*** J.A. Allen, 1917. English: Russet Free-tailed Bat. Chinese: 赤犬吻蝠. Recorded from five widely separated localities in West, Central and East Africa, from Côte d’Ivoire to Kenya. Mostly in Guinea woodlands at the edge of rainforests (Happold M, 2013za as *Tadarida russata*). In Kenya, recorded from Hell’s Gate Canyon in the mosaic of East African evergreen bushland and secondary *Acacia* wooded grassland.

**Genus** ***Mops*** Lesson, 1842. Free-tailed Bats

231. ***Mops brachypterus*** (Peters, 1852). English: Short-winged Free-tailed Bat. Chinese: 短翼犬吻蝠. Disjunct records from West Africa, NE DR Congo and Uganda, and from SE Kenya to NE Mozambique (including Zanzibar and Mozambique islands). Found in lowland rainforests, rainforest-ecotone, rainforest and secondary grassland mosaics and coastal forest mosaics (Happold M, 2013zb as *Tadarida brachyptera*). In Kenya, recorded only from the SE (coastal strip).
232. ***Mops condylurus*** (A. Smith, 1833). English: Angolan Free-tailed Bat. Chinese: 安哥拉犬吻蝠. Widespread but disjunct records from much of sub-Saharan Africa. Mostly found in woodland savannas but also in rainforest and secondary grassland mosaics and coastal mosaics (Happold M, 2013zc as *Tadarida condylura*). In Kenya, recorded widely in the W and S and along the coastal strip.
233. ***Mops midas*** (Sundevall, 1843). English: Midas Free-tailed Bat. Chinese: 米达犬吻蝠. Widespread but highly disjunct records in sub-Saharan Africa. Mostly found in woodland savannas close to rivers and wetlands (Cotterill & Happold M, 2013e as *Tadarida midas*). In Kenya, recorded at one locality in the NW (Freeman, 1981).
234. ***Mops nanulus*** J.A. Allen, 1917. English: Dwarf Free-tailed Bat. Chinese: 侏犬吻蝠. Disjunct records from several countries in West Africa, as well as Central and eastern Africa, including S Sudan, W Ethiopia, Uganda and Kenya. Mostly found in various habitats near the edges of lowland rainforests, but occasionally in woodland savannas and riverine forests (Happold M, 2013zd as *Tadarida nanula*). In Kenya, recorded only in the W.
235. ***Mops thersites*** (Thomas, 1903). English: Railer Free-tailed Bat. Chinese: 无畏犬吻蝠. Somewhat disjunct records in small parts of West, Central and East Africa (excluding Tanzania). Found in lowland rainforests and secondary forests, invasive Guinea woodland savannas, and rainforest and secondary grassland mosaics (Happold M, 2013ze as *Tadarida thersites*). In Kenya, recorded only from the SW.

**Genus** ***Otomops*** Thomas, 1913. Giant Mastiff Bats

236. ***Otomops harrisoni*** Ralph, Richards, Taylor, Napier & Lamb, 2015. English: Harrison’s Giant Mastiff Bat. Chinese: 哈氏巨犬吻蝠. *Otomops harrisoni* was previously included in *Otomops martiensseni*. Disjunct records in Djibouti, Ethiopia and Kenya in a wide range of habitats (Ralph et al., 2015). In Kenya, found in a narrow central band from the NW to SE (Yalden & Happold M, 2013 as *Otomops martiensseni*).
237. ***Otomops martiensseni*** (Matschie, 1897). English: Large-eared Giant Mastiff Bat. Chinese: 大耳犬吻蝠. Widely scattered localities from Guinea-Bissau to Kenya, Uganda and south to Angola and South Africa. In Kenya, known with certainty only from forests in Marsabit but may occur broadly in Kenya (Patterson et al., 2018).

**Genus** ***Platymops*** Thomas, 1906. Peters’s Flat-headed Bat

238. ***Platymops setiger*** (Peters, 1878). English: Peters’s Flat-headed Bat. Chinese: 彼德犬吻蝠. Recorded from S Sudan and SW Ethiopia in a narrow band to SE Kenya. Mostly found in *Acacia*-*Commiphora* deciduous bushland and thickets, and mosaics of East African evergreen bushland and secondary grassland (Happold M, 2013zf). In Kenya, recorded in a wide band from Turkana in the NW through the Nuu Hills, Kitui, Makindu and Kibwesi to the Taita Hills in the SE, mostly from dry stony areas and areas with rocky hills (Aggundey & Schlitter, 1984; Happold M, 2013zf).

**Genus** ***Tadarida*** Rafinesque, 1814.Free-tailed Bats

239. ***Tadarida aegyptiaca*** (É. Geoffroy, 1818). English: Egyptian Free-tailed Bat. Chinese: 北非犬吻蝠. Recorded from widespread but very disjunct localities in Africa (including North-West and North-East Africa and the Sahara, C Nigeria and eastern Africa) but with most records from southern Africa. Mostly found in open woodland and bushland savannas (Bernard & Happold M, 2013d). Found in some very arid areas but probably only where drinking water, insects and suitable day-roosts are available. In Kenya, recorded in the SE.
240. ***Tadarida fulminans*** (Thomas, 1903). English: Madagascan Free-tailed Bat. Chinese: 岛犬吻蝠. Recorded from several disjunct areas and localities from Kenya to Zimbabwe in woodland savannas (Cotterill, 2013d). In Kenya, recorded only from the mid-W and mid-SW.
241. ***Tadarida lobata*** (Thomas, 1891). English: Big-eared Free-tailed Bat. Chinese: 大耳犬吻蝠. Highly disjunct records only from Kenya and Zimbabwe. Found in woodland savannas (Cotterill, 2013e). In Kenya, recorded from several localities in the W, and from one locality in the SE (in flat open thorn scrubland with scattered rocky hills, including Maungu Hill) (Cotterill, 2013e).
242. ***Tadarida ventralis*** (Heuglin, 1861). English: Giant Free-tailed Bat. Chinese: 非洲大犬吻蝠. Recorded from several localities in eastern and southern Africa, from Eritrea and Ethiopia to E Zambia, W Mozambique, S Malawi, Zimbabwe and NE South Africa (but not most of Tanzania and Mozambique). Mostly found in dry woodland savannas but also in some montane habitats (Cotterill, 2013f). Most records are from Kenya. In Kenya, widely recorded except in the NE and E, in the Kenyan Highlands, semi-desert grassland and shrubland near Lake Turkana, and *Acacia-Commiphora* bushland (Cotterill, 2013f).

**Family MINIOPTERIDAE**

Previously considered to be a subfamily of Vespertilionidae (see also Simmons, 2005), but now recognized as a valid family (e.g., Hoofer & Van Den Bussche, 2003; Miller-Butterworth et al., 2007).

**Genus** ***Miniopterus*** Bonaparte, 1837. Long-fingered Bats

243. ***Miniopterus africanus*** Sanborn, 1936. English: African Long-fingered Bat. Chinese: 非洲长翼蝠. Previously considered a subspecies of *M. inflatus*, but its specific status was confirmed by Juste et al. (2007). Recorded from Eritrea, Ethiopia, Kenya and Tanzania in various dry habitats. In Kenya, recorded from the W, S and SE and in the Rift Valley and comparatively dry savanna habitats (Happold M, 2013zg as *M. i. africanus*).
244. ***Miniopterus fraterculus*** Thomas and Schwann, 1906. English: Lesser Long-fingered Bat. Chinese: 小长翼蝠. Disjunct records from E DR Congo, Kenya, Tanzania and W Zambia, and contiguously from NE Zimbabwe and S Malawi to S Mozambique and the coastal belt of South Africa (Bernard & Happold M, 2013e). In Kenya, recorded from the SW and SE, mostly in *Acacia-Commiphora* deciduous bushland and thickets.
245. ***Miniopterus inflatus*** Thomas, 1903. English: Greater Long-fingered Bat. Chinese: 大长翼蝠. Recorded from very isolated and small localities in sub-Saharan Africa, from Liberia to Kenya and south to N Nambia, Zimbabwe, Malawi and Mozambique. Found in dry savanna habitats, *Acacia* scrubs, montane forests and lowland rainforests (Happold M, 2013zh). In Kenya, recorded from a narrow area in the W-SW in wetter habitats than that of *M. africanus*.
246. ***Miniopterus minor*** Peters, 1867. English: Least Long-fingered Bat. Chinese: 侏长翼蝠. Recorded from very isolated localities in the Congo (near Congo R.) and DR Congo, and also from the coastal strip near the Kenya-Tanzania border. Western records are from woodland savanna near caves, eastern records are from coastal savanna and forest mosaics (Happold M, 2013zi). In Kenya, recorded only from SE and N of Mombasa.
247. ***Miniopterus mossambicus*** Monadjem, Goodman, Stanley & Appleton, 2013. English: Mozambican Long-fingered Bat. Chinese: 莫桑比克长翼蝠. Recorded from Mozambique and Taita Hills in Kenya in a wide variety of habitats (López-Baucells et al., 2016; Monadjem et al., 2013c).
248. ***Miniopterus natalensis*** (A. Smith, 1834). English: Natal Long-fingered Bat. Chinese: 纳塔尔长翼蝠. Recorded widely in eastern, south-central and southern Africa, from South Sudan and Ethiopia, and from Kenya southwards through part of Tanzania, to Zambia, Zimbabwe, Namibia, NE Botswana, S Mozambique and parts of South Africa; in various habitats but not forests (Bernard & Happold M, 2013f). In Kenya, currently recorded in a band from the mid-W to SE, mostly in *Acacia-Commiphora* deciduous bushland and thickets. However, identification/affinities of specimens from wetter habitats (including afro-montane vegetation and forest-savanna mosaics) in the W and SE need confirmation (Bernard & Happold M, 2013f).

**Family VESPERTILIONIDAE**

**Genus** ***Eptesicus*** Rafinesque, 1820. Serotines

249. ***Eptesicus hottentotus*** (A. Smith, 1833). English: Long-tailed Serotine. Chinese: 长尾棕蝠. Disjunct records from Kenya, N Zambia, and much of Central and southern Africa (excluding Botswana). Found in woodland savannas and along rivers with permanent water in deserts (Cotterill & Happold M, 2013f). In Kenya, recorded only from C areas (Naivasha, Hell’s Gate) in rocky gorges near water.

**Genus** ***Glauconycteris*** Dobson, 1875. Butterfly Bats

250. ***Glauconycteris argentata*** (Dobson, 1875). English: Common Butterfly Bat. Chinese: 银蝶蝠. Disjunct records in Central and East Africa, from Cameroon and N Angola to Kenya, Tanzania and N Malawi. Found in rainforest habitats, miombo woodland and coastal forests (Happold M, 2013zj). In Kenya, recorded only from the W (including near Kakamega Forest).
251. ***Glauconycteris humeralis*** J.A. Allen, 1917. English: Spotted Butterfly Bat. Chinese: 花蝶蝠. Recorded from a narrow band of localities extending from N DR Congo to Uganda and W Kenya, and from one locality in E DR Congo. Found in rainforests and rainforest-savanna mosaics (Eger & Schlitter, 2001; Happold M, 2013zk; Heller et al., 1994). In Kenya, recorded from Kakamega Forest in the W, where there are numerous grassy glades in extensive stands of tall evergreen forest.
252. ***Glauconycteris kenyacola*** Peterson, 1982. English: Kenyacola Butterfly Bat. Chinese: 肯尼亚蝶蝠. Endemic to Kenya. As yet, recorded only from coastal forest at the mouth of Tana River, where it is known only from its type specimen (Happold M, 2013zl).
253. ***Glauconycteris variegata*** (Tomes, 1861). English: Variegated Butterfly Bat. Chinese: 彩蝶蝠. Widespread but highly disjunct records in sub-Saharan Africa. Mostly found in savanna habitats, including woodland savannas and open bush country (Happold M, 2013zm). In Kenya, recorded in the W (Kakamega Forest) but also from Garissa (Aggundey & Schlitter, 1984).

**Genus** ***Hypsugo*** Kolenati, 1856. Pipistrelle Bats

Van Cakenberghe & Happold M (2013b) provisionally treated *Hypsugo* and all other African pipistrelles as members of the genus *Pipistrellus*. We followed Monadjem et al. (2013a) in treating *Hypsugo* as distinct.

254. ***Hypsugo crassulus*** (Thomas, 1904). English: Broad-headed Pipistrelle. Chinese: 宽首伏翼. Disjunct records from Guinea, Liberia and Côte d’Ivoire in West Africa (*H. c. bellieri*) and from SW Cameroon, Congo, NE DR Congo, South Sudan, Uganda, W Kenya and NE Angola (*H. c. crassulus*). Found in lowland rainforests, swamps, and coastal and montane forests (Fahr, 2013f as *Pipistrellus crassulus*). In Kenya, recorded only from the W (Rondo in Kakamega Forest).

**Genus** ***Kerivoula*** Gray, 1842. Woolly Bats

255. ***Kerivoula argentata*** Tomes, 1861. English: Damara Woolly Bat. Chinese: 银彩蝠. Disjunct records from some parts of Central, eastern and southern Africa. Found in evergreen and riverine forests, and both mesic and dry woodland savannas (Cotterill, 2013g). Two records from Angola need confirmation. In Kenya, recorded from the SE.
256. ***Kerivoula lanosa*** (A. Smith, 1847). English: Lesser Woolly Bat. Chinese: 小彩蝠.Disjunct records from Liberia and Guinea to Ethiopia, and from E DR Congo and Kenya southwards to South Africa. Found in a wide variety of habitats (Cotterill, 2013h). In Kenya, recorded in C regions and parts of the E and S (Cotterill, 2013h).
257. ***Kerivoula smithii*** Thomas, 1880. English: Smith’s Woolly Bat. Chinese: 史密斯彩蝠. Disjunct records in a narrow band from SE Nigeria, Cameroon, N and NE DR Congo and Uganda to Kenya. Found in lowland rainforests, swamp forests, mangroves, and montane and riverine forests (Fahr, 2013g). In Kenya, recorded from the E Aberdare Ranges and Bura (near Garissa) on the Tana River.

**Genus** ***Laephotis*** Thomas, 1901. African Long-eared Bats

258. ***Laephotis wintoni*** Thomas, 1901. English: De Winton’s Long-eared Bat. Chinese: 温氏长耳蝠. Highly disjunct records from Ethiopia, Kenya, Tanzania and South Africa. Found in a wide variety of habitats (Kearney, 2013a). In Kenya, recorded in a narrow band from C to CS regions (Nanyuki, Samburu, Nyeri, Kitui and Namanga).

**Genus** ***Mimetillus*** Thomas, 1904. Moloney’s Mimic Bat

259. ***Mimetillus moloneyi*** (Thomas, 1891). English: Moloney’s Mimic Bat. Chinese: 非洲扁颅蝠. Disjunct records in sub-Saharan Africa from Sierra Leone to W Ethiopia and Kenya and S to Angola, Zambia and Mozambique. Found along the edges of rainforests and in forest-savanna mosaics, woodlands and coastal forests (Fahr, 2013h). In Kenya, recorded disjunctly from the W, E and S.

**Genus** ***Myotis*** Kaup, 1829. Mouse-eared Bats

260. ***Myotis bocagii*** (Peters, 1870). English: Rufous Myotis. Chinese: 棕红鼠耳蝠. Disjunct records in sub-Saharan Africa (except the Horn of Africa) and from C Angola, Namibia, Zimbabwe to most of South Africa. Found in lowland rainforests, rainforest-savanna mosaics, and woodland savannas and coastal forests, but probably only within reach of open water (Happold M, 2013zn). In Kenya, recorded from the SW.
261. ***Myotis tricolor*** (Temminck, 1832). English: Temminck’s Myotis. Chinese: 南非鼠耳蝠. Widespread but highly diisjunct records in sub-Saharan Africa from Ethiopia to South Africa in the eastern half of the continent, and from two isolated localities in Senegal and SW DR Congo. Found in a wide variety of habitats. In Kenya, recorded from the W to C and towards the SE but not reaching the coast; in rainforest, montane forest and *Acacia-Commiphora* deciduous bushland and thickets (Bernard, 2013).
262. ***Myotis welwitschii*** (Gray, 1866). English: Welwitsch’s Myotis. Chinese: 魏氏鼠耳蝠. Disjunct records from Ethiopia to South Africa, and isolated localities in Guinea, Cameroon and N Angola. Found in a wide variety of habitats, often close to mountains but not confined to high altitudes (Happold M, 2013zo). In Kenya, recorded in the W (including in Nandi, Kakamega, Kisumu) in montane forests.

**Genus** ***Nycticeinops*** Hill and Harrison, 1987. Schlieffen’s Twilight Bat

263. ***Nycticeinops schlieffeni*** (Peters, 1859). English: Schlieffen’s Twilight Bat. Chinese: 施氏墓蝠. Widespread but disjunct records from S Mauritania to Sudan (with outlying localities in N Egypt), and southwards (mostly on E side of continent) to NE South Africa. Found in semi-arid grasslands, shrublands, various woodlands and some coastal habitats (Happold M, 2013zp). In Kenya, recorded very widely from the NW to S and E, mostly in *Acacia-Commiphora* deciduous bushland and thickets and semi-arid grassland.

**Genus** ***Neoromicia*** Roberts, 1926. Pipistrelle Bats

Van Cakenberghe & Happold M (2013b) provisionally treated *Neoromicia* and all other African pipistrelles as members of the genus *Pipistrellus*. Here we followed Goodman et al. (2012) and Monadjem et al. (2013a) in treating them as distinct genera.

264. ***Neoromicia capensis*** (A. Smith, 1829). English: Cape Pipistrelle. Chinese: 南非伏翼. Recorded very widely (but with large gaps) in sub-Saharan Africa from West Africa to Eritrea and southwards to South Africa. Found in most vegetation zones, except large deserts and some coastal habitats (Kearney, 2013b as *Pipistrellus capensis*). In Kenya, widespread.
265. ***Neoromicia*** cf. ***helios*** auctorum non Heller, 1912. English: Samburu Pipistrelle. Chinese: 桑布鲁伏翼. The taxonomy of cf. *helios* is uncertain. It is not yet known whether or not some Kenyan bats referred to as *helios* (e.g., by Hill & Harrison, 1987) or studied in Kenya by O’Shea (1980) as *Pipistrellus nanus* belong to a species that is distinct from the type of *helios* (Heller, 1912) (Happold M & Van Cakenberghe, 2013 as *Pipistrellus* cf. *helios*). Many characteristics distinguish cf. *helios* from *Neoromicia nana*, including roosting behavior, social behavior, tail glands, bacular morphology and lower molars. However, the confusion between *helios*, cf. *helios* and *nana* calls for further molecular investigation. Recorded in eastern Africa from S Somalia, S Sudan, Uganda, Kenya and NE Tanzania, with an outlying record from Djibouti needing confirmation. Found in a variety of habitats (Happold M & Van Cakenberghe, 2013 as *Pipistrellus* cf. *helios*). In Kenya, widespread except in the N and NE, mostly in *Acacia-Commiphora* deciduous bushland and thickets and semi-desert grassland and shrubland, but also in montane vegetation, rainforest and secondary grassland mosacis, and East African coastal forest mosaics.
266. ***Neoromicia nana*** (Peters, 1852). English: Banana Pipistrelle. Chinese: 香蕉伏翼. Recorded throughout most of sub-Saharan Africa (except the Horn of Africa and the SW, including most of Namibia, Botswana, W Zimbabwe and South Africa). Found in diverse forests, savanna and sub-desert steppe habitats, but possibly only where banana plants and/or other musaceous plants are found (Happold M, 2013zq as *Pipistrellus nanus*). In Kenya, recorded only in a narrow band along the Kenya-Tanzania border from Uganda to the coastal strip.
267. ***Neoromicia rendalli*** (Thomas, 1889). English: Rendall’s Pipistrelle. Chinese: 任氏伏翼. Disjunct records in sub-Saharan Africa from Senegal to S Somalia and southwards to South Africa, but not in all countries. Mostly found in woodland savannas and degraded lowland rainforest habitats on the rainforest zone border (Van Cakenberghe & Happold M, 2013c as *Pipistrellus rendalli*). In Kenya, recorded in some C to SE areas, mostly in *Acacia-Commiphora* deciduous bushland and thickets.
268. ***Neoromicia somalica*** (Thomas, 1901). English: Somali Pipistrelle. Chinese: 索马里伏翼. Recorded in sub-Saharan Africa from Gambia to Djibouti, N and S Somalia, Kenya and N Tanzania, with isolated records from Congo and DR Congo. Mostly found in savanna habitats, rainforest-savanna mosaics, bushlands, riverine forests and the East African coastal forest mosaics (Van Cakenberghe & Happold M, 2013d as *Pipistrellus somalicus*). Possibly also occurs southwards to Namibia, Botswana, Mozambique and South Africa but this needs confirmation because of confusion between *N. somalica* and *N. zuluensis* (Van Cakenberghe & Happold M, 2013d as *Pipistrellus somalicus*). In Kenya, recorded from the SW to SE and along the coastal strip, mostly in areas of dense thorn scrub (dominated by *Combretum*, *Commiphora* and *Acacia*) with scattered patches of grassland, but also in riverine woodland along the Athi River and in coastal forest mosaics.
269. ***Neoromicia tenuipinnis*** (Peters, 1872). English: White-winged Pipistrelle. Chinese: 白翼伏翼. Disjunct records in sub-Saharan Africa from West Africa to eastern Africa and some parts of Central Africa. Mostly found in lowland rainforests and swamp and coastal forests, but also in mangroves, montane forests, forest-savanna mosaics and various woodland savannas (Fahr, 2013i as *Pipistrellus tenuipinnis*). In Kenya, recorded only from the SW.
270. ***Neoromicia zuluensis*** (Roberts, 1924). English: Zulu Pipistrelle. Chinese: 祖鲁伏翼. *Neoromicia zuluensis* was formerly treated as a synonym of *N. somalica* (e.g., Koopman, 1984). However, karyotypic data confirmed *zuluensis* as distinct from *somalica* (Rautenbach et al., 1993), though some records in East Africa may reflect the former synonymy of these taxa. Additionally, while much of the literature indicates that isolated populations of *N. zuluensis* occur in dry savanna habitats of *Acacia-Commiphora* deciduous bushland and thickets in Ethiopia, S. Sudan, E Uganda, W and E Kenya and southern Africa (e.g., Happold M et al., 2013), doubt exists as to whether the species occurs N of southern Africa from where the type specimen comes. Therefore, a comprehensive revision of the two species in eastern Africa is required.

**Genus** ***Pipistrellus*** Kaup, 1829. Pipistrelle Bats

271. ***Pipistrellus aero*** Heller, 1912. English: Mt. Gargues Pipistrelle. Chinese: 东非伏翼. Recorded from three widely separated localities in Ethiopia (Lavrenchenko et al., 2004) and from Kenya (Van Cakenberghe & Happold M, 2013e). In Kenya, recorded in the N, C and S at Mt. Gargues, Lake Marsabit and Ngong (Aggundey & Schlitter, 1984); in montane forests, evergreen bushland and secondary *Acacia*-wooded grassland mosacis, and near afro-montane vegetation.
272. ***Pipistrellus grandidieri*** (Dobson, 1876). English: Yellow Pipistrelle. Chinese: 黄伏翼. Recorded from isolated localities in Cameroon, Uganda, Burundi, S Somalia, SE Kenya, NE coast of Tanzania, Zanzibar Island, C Angola and S Malawi. Found in montane forests, riverine habitats and woodlands (Van Cakenberghe & Happold M, 2013f). In Kenya, recorded only in the extreme SE in coastal forests.
273. ***Pipistrellus hesperidus*** (Temminck, 1840). English: Dusk Pipistrelle. Chinese: 暗黑伏翼. Recorded from Cameroon and Bioko Island (and perhaps Liberia and Côte d’Ivoire), further away in Angola and W Zambia, and from Eritrea to South Africa and N Somalia. Found in diverse habitats (Kearney, 2013c). In Kenya, recorded in C and from SW to SE regions along the Kenya-Tanzania border (Aggundey & Schlitter, 1984 as *Pipistrellus kuhlii fuscatus*) in diverse habitats.
274. ***Pipistrellus nanulus*** Thomas, 1904. English: Tiny Pipistrelle. Chinese: 中非小伏翼. Disjunct records in western and Central Africa (from Senegal to Cameroon, Bioko Island and Gabon) and also in E DR Congo, Uganda and Kenya. Found in rainforests, woodland savannas and riverine habitats (Van Cakenberghe & Happold M, 2013g). In Kenya, recorded only in the W near Kakamega Forest.
275. ***Pipistrellus rueppellii*** (Fischer, 1829). English: Rüppell’s Pipistrelle. Chinese: 吕氏伏翼. Widespread but disjunct records from most African countries. Mostly found in woodland and grassland savannas but occasionally in desert habitats, montane forests and along some river systems in rainforest zones (Happold M, 2013zr). In Kenya, recorded only from the SW.
276. ***Pipistrellus rusticus*** (Tomes, 1861). English: Rustic Pipistrelle. Chinese: 锈色伏翼. Disjunct records from Senegal and Gambia to Ethiopia (but not in all countries) and from W Kenya, E Tanzania, S Zambia, Namibia, Botswana, Zimbabwe and NE South Africa. Found in montane and riverine forests, woodland and dry savannas, and coastal forest and scrub (Kearney, 2013d). In Kenya, only recorded from the mid-W on the border with Uganda.

**Genus** ***Scotoecus*** Thomas, 1901. Lesser House Bats

In Africa, there are light-winged and dark-winged forms. The taxonomy of the four dark-winged forms recognized by Hill (1974) needs revision because sexual dimorphism was not considered when they were distinguished and, in some cases, subsequently placed in distinct species (Happold M, 2013zs). Aggundey & Schlitter (1984) referred to extensive records of *Scotoecus hindei hindei* and *S. h. albigula* from across Kenya. Monadjem et al. (2010) grouped *S. albigula* and *S. hindei* as they could not differentiate them. They suggested that the two species were probably conspecific, but this requires genetic/molecular confirmation. Until this taxonomic issue is resolved, we only recognized one dark-winged species.

277. ***Scotoecus albofuscus*** (Thomas, 1890). English: Light-winged Lesser House Bat. Chinese: 淡翼宽吻蝠. Recorded from widespread localities from Gambia to Cameroon in West Africa, and from Uganda and Kenya to NE South Africa (but not in all countries within these ranges). Found in woodland savannas, grassland and thicket mosaics, and in various forests and woodlands (but not in rainforest zones) (Happold M, 2013zt). In Kenya, recorded only from the SE in the East African coastal mosaics.
278. ***Scotoecus hirundo*** (de Winton, 1899). English: Dark-winged Lesser House Bat. Chinese: 暗翼宽吻蝠. Recorded from many widespread but disjunct localities from Senegal to Sudan, Ethiopia and S Somalia and southwards (mostly on the E side of continent) to E Angola, Zambia and S Malawi. Mostly found in open woodlands in West Africa, in woodlands and deciduous thicket and bushland in the E, and in wetter and drier miombo woodland in the S (Happold M, 2013zu). In Kenya, widespread except in the N and E.

**Genus** ***Scotophilus*** Leach, 1821. Yellow house Bats

Many Kenyan records of yellow house bats have traditionally been referred to either *Scotophilus dinganii* or *S. viridis*. Both taxa were originally described from southern Africa. Trujillo et al. (2009) showed that these Kenyan bats clearly belonged to clades that differed from typical *S. dinganii* and *S. viridis*. Brooks & Bickham (2014) proposed four new species for clades defined by Trujillo et al. (2009) but failed to distinguish their new taxa from various older names, especially *colias* Thomas, 1904, which has a type locality of Fort Hall (Muranga) in Kenya.
279. ***Scotophilus andrewewborii*** Brooks & Bickham, 2014. English: Andrew Rebori’s House Bat. Chinese: 安氏黄蝠. Formerly listed as *S. dinganii*. As yet, only recorded from Kenya where it is widespread from the W of the country to coastal areas, in a wide range of savanna and woodland habitats (Brooks & Bickham, 2014).
280. ***Scotophilus leucogaster*** (Cretzschmar, 1830). English: White-bellied House Bat. Chinese: 白腹黄蝠. Disjunct records from West Africa to Sudan and W Ethiopia and southwards to southern Africa (but not all countries). Found in a wide variety of habitats (Van Cakenberghe & Happold M, 2013h). In Kenya, recorded from four disjunct localities in the NW, NC, SW and SE.
281. ***Scotophilus livingstonii*** Brooks & Bickham, 2014. English: Livingstone’s House Bat. Chinese: 利文斯顿黄蝠. Formerly listed as *S. dinganii*. Known from W Kenya and Ghana (Brooks & Bickham, 2014).
282. ***Scotophilus nigrita*** (Schreber, 1774). English: Giant House Bat. Chinese: 非洲大黄蝠. Highly disjunct localites in West Africa (Côte d’Ivoire to SW Nigeria) and in Sudan, SE DR Congo, SE Kenya, NE Tanzania, S Malawi, E Zimbabwe and S Mozambique. Mostly found in dry woodland savannas near rivers and riverine forests (Happold M, 2013zv). The apparently disjunct distribution might reflect inadequate collection of this hard-to-catch species. In Kenya, recorded only from the Shimba Hills in the SE in coastal forest mosaics.
283. ***Scotophilus nux*** Thomas, 1904. English: Nut-coloured House Bat. Chinese: 喀麦隆黄蝠. Disjunct records from Sierra Leone to SE Nigeria, SW Nigeria and Cameroon, and NW DR Congo to W Kenya. Found in lowland rainforests and rainforest clearings (and swamp forests in Côte d’Ivoire) (Van Cakenberghe & Happold M, 2013i). In Kenya, recorded only from the SW in forests near the Kakamega Forest.
284. ***Scotophilus trujilloi*** Brooks & Bickham, 2014. English: Trujillo’s House Bat. Chinese: 特鲁黄蝠. Formerly listed as *S. viridis* (Van Cakenberghe & Happold M, 2013j). Recorded only from SE Kenya (Shimba Hills NP and Taita District) Brooks & Bickham, 2014), but probably also occurs in NE Tanzania. Found in woodland and coastal forest habitats and human-modified habitats.

**ORDER CARNIVORA (Carnivorans–36 Species)**

**Family CANIDAE**

**Genus** ***Canis*** Linnaeus, 1758. Jackals and Wolves 

The Golden Jackal is restricted to Asia and does not occur in Africa. Recent taxonomy on Golden Jackals (Atickem et al., 2017; Gaubert et al., 2012; Viranta et al., 2017) considers the entire African “golden jackal” group to be the African Wolf (*Canis lupaster*). In addition, Gaubert et al. (2012) suggested that an African Golden Jackal (distinct from both the Asian Golden Jackal and *C. lupaster*) may exist in Africa, but there is no clear evidence for this.

285. ***Canis lupaster*** Hemprich and Ehrenberg, 1832. English: African Wolf. Swahili: Mbweha. Chinese: 非洲狼. Previously known as *Canis aureus* (Moehlman & Jhala, 2013), though the name was recently changed to *C. lupaster* (Atickem et al., 2017; Viranta et al., 2017) after molecular review. Recorded from northern Africa, including Senegal to Morocco through to the Horn of Africa as well as East Africa (Moehlman & Jhala, 2013 as *C. aureus*). In desert and semi-desert habitats as well as savanna woodlands. In Kenya, confirmed records in the Solio Ranch in the SE Laikipia County as well as in the N part of the country.

**Genus** ***Lupulella*** Hilzheimer, 1906. African Jackals

Following Viranta et al. (2017) and Atickem et al. (2017), we recognise the genus *Lupulella*.

286. ***Lupulella adusta*** Sundevall, 1847. English: Side-striped Jackal. Swahili: Mbweha Miraba. Chinese: 侧纹胡狼. Formerly referred to as *Canis adustus*; however, recent molecular review of the genus placed the species within *Lupulella* (Atickem et al., 2017; Viranta et al., 2017). Recorded from many African countries S of the Sahara. In a wide variety of habitats. In Kenya, widely distributed (Loveridge & Macdonald, 2013 as *Canis adustus*).
287. ***Lupulella mesomelas*** Schreber, 1775. English: Black-backed Jackal. Swahili: Mbweha Shaba/Nyekundu/Fedha. Chinese: 黑背胡狼. Formerly referred to as *Canis mesomelas*, but now placed within the genus *Lupulella.* Two isolated population ranges of two subspecies: *Canis m. mesomelas* in southern African and *Canis m. schmidti* in East Africa and the Horn of Africa. In a wide variety of habitats. In Kenya, widely distributed (Loveridge & Nel, 2013 as *Canis m. mesomelas*).

**Genus** ***Lycaon*** Brookes, 1827. African Wild Dog

288. ***Lycaon pictus*** (Temminck, 1820). English: African Wild Dog: Swahili: Mbwa Mwitu. Chinese: 非洲野犬. Recorded from North, East and southern Africa and Ethiopia. In short-grass plains, semi-deserts, bushy savannas and upland forests. In Kenya, recorded from the S (Tsavo East and West NPs), E and N (some Laikipia ranches) (McNutt & Woodroffe, 2013).

**Genus** ***Otocyon*** Müller, 1835. Bat-eared Fox

289. ***Otocyon megalotis*** (Desmarest, 1822). English: Bat-eared Fox. Swahili: Mbweha Masikio. Chinese: 大耳狐. Two subspecies with isolated population ranges: *Otocyon m. megalotis* recorded from southern Africa and *Otocyon m. virgatus* in East Africa, including Ethiopia, Somalia and South Sudan. In open grasslands, especially short-grass plains, open scrub vegetation, arid and semi-arid areas and open arid savannas. In Kenya, widely distributed (Nel & Mass, 2013).

**Family MUSTELIDAE**

**Genus** ***Ictonyx*** Kaup 1835. Zorilla

290. ***Ictonyx striatus*** (Perry, 1810). English: Zorilla. Swahili: Kicheche. Chinese: 非洲艾虎. Widespread in many African countries S of the Sahara. In a wide variety of habitats. In Kenya, widely distributed (Stuart & Stuart, 2013a).

**Genus** ***Poecilogale*** Thomas, 1883. Africa Striped Weasel

291. ***Poecilogale albinucha*** (Gray, 1864). English: Africa Striped Weasel. Swahili: Chororo. Chinese: 白颈鼬. Recorded from S of the Equator, from SW Uganda and Kenya to the Western Cape in South Africa. In savanna habitat associations. In Kenya, recorded from the SW-W (Stuart & Stuart, 2013b).

**Genus** ***Aonyx*** Lesson, 1827. Clawless Otters

292. ***Aonyx capensis*** (Schinz, 1821). English: African Clawless Otter. Swahili: Fisi Maji Mkubwa. Chinese: 非洲小爪水獭. Widespread in many African countries S of the Sahara. In freshwater aquatic habitats but also in marine habitats where fresh water is accessible (Somers & Nel, 2013). In Kenya, recorded mainly W of the Rift Valley.

**Genus** ***Hydrictis*** Pocock, 1921. Spotted-necked Otter

293. ***Hydrictis maculicollis*** Lichtenstein, 1835. English: Spot-necked Otter. Swahili: Fisi Maji Mdogo. Chinese: 斑颈水獭. Recorded from West, Central, East and southern Africa, including Ethiopia and South Sudan. In freshwater habitats where water is unstilted, unpolluted, and rich in small fish. In Kenya, recorded in the S and W (Carranza & Rowe-Rowe, 2013).

**Genus** ***Mellivora*** Storr, 1780. Ratel

294. ***Mellivora capensis*** (Schreber, 1776). English: Ratel, Honey Badger. Swahili: Nyegere. Chinese: 蜜獾. Very widespread in Africa, except in some countries in North Africa. In a wide variety of habitats from deserts to moist rainforests. In Kenya, widely distributed (Begg et al., 2013).

**Family NANDINIIDAE**

**Genus** ***Nandinia*** Gray, 1843. Two-spotted Palm Civet

295. ***Nandinia binotata*** (Gray, 1830). English: Two-spotted Palm Civet. Swahili: Ngawa. Chinese: 双斑椰子猫. Recorded from West to East Africa, including Malawi, W Mozambique, E Zimbabwe and NE Zambia. In deciduous forests, lowland rainforests and mountains. In Kenya, recorded W of the Rift Valley and in some C regions (Van Rompaey & Ray, 2013).

**Family FELIDAE**

**Genus** ***Panthera*** Oken, 1816. Roaring Cats

296. ***Panthera leo*** (Linnaeus, 1758). English: Lion. Swahili: Simba. Chinese: 狮. Recorded from many countries in Africa S of the Sahara. In all habitat types, except in rainforests and the interior of the Sahara Desert. In Kenya, recorded in the N, S and E (West & Packer, 2013).
297. ***Panthera pardus*** (Linnaeus, 1758). English: Leopard. Swahili: Chui. Chinese: 豹. Recorded from many countries in sub-Saharan Africa. In a wide variety of habitats. In Kenya, widely distributed (Hunter et al., 2013).

**Genus** ***Profelis*** Severtzov, 1858. African Golden Cat

298. ***Profelis aurata*** (Temminck, 1827). English: African Golden Cat. Swahili: Paka. Chinese: 非洲金猫. Recorded from West Africa, Central to East Africa, including South Sudan and Ethiopia. In undisturbed and disturbed tropical forests. Widespread in S Kenya (Ray & Butynski, 2013).

**Genus** ***Caracal*** Gray, 1843. Caracal

299. ***Caracal caracal*** (Schreber, 1776). English: Caracal. Swahili: Simba Mangu. Chinese: 狞猫. Recorded in many countries in Africa both N and S of the Sahara. In semi-deserts to relatively open savanna, scrubland to moist woodlands and thickets. In Kenya, widely distributed (Stuart & Stuart, 2013c).

**Genus** ***Leptailurus*** Severtzov, 1858. Serval

300. ***Leptailurus serval*** (Schreber, 1776). English: Serval. Swahili: Mondo. Chinese: 薮猫. Recorded from many African countries S of the Sahara. In a wide variety of habitats with permanent water sources. In Kenya, widely distributed (Hunter & Bowland, 2013).

**Genus** ***Acinonyx*** Brookes, 1828. Cheetah

301. ***Acinonyx jubatus*** Schreber, 1775. English: Cheetah. Swahili: Duma. Chinese: 猎豹. Recorded from southern, eastern and northern Africa. In open plains and miombo savanna woodlands. In Kenya, recorded in the S and N (Caro, 2013).

**Genus** ***Felis*** Linnaeus, 1758. Small Cats

302. ***Felis silvestris*** Schreber, 1777. English: Wildcat. Swahili: Paka Witu/Paka Pori/Kimburu. Chinese: 欧林猫. Widely distributed in Africa, except in some parts of Central and North Africa. In a wide variety of habitats. In Kenya, widely distributed (Stuart et al., 2013).

**Family VIVERRIDAE**

**Genus** ***Genetta*** Cuvier, 1816. Genets

303. ***Genetta genetta*** (Linnaeus, 1758). English: Common Genet. Swahili: Kanu Mdogo Madoa. Chinese: 小斑獛. Recorded from southern, West, East, Central and North Africa and the Horn of Africa. In all types of wooded habitats (deciduous and evergreen). In Kenya, widely distributed (Delibes & Gaubert, 2013).
304. ***Genetta maculata*** (Gray, 1830). English: Large-spotted Genet. Swahili: Kanu. Chinese: 斑獛. Recorded from many African countries S of the Sahara. In a wide variety of forested habitats. In Kenya, widely distributed (Angelici & Gaubert, 2013).
305. ***Genetta servalina*** Pucheran, 1855. English: Servaline Genet. Swahili: Kanu. Chinese: 尼日利亚獛. Recorded from Central to East Africa. In primary and secondary lowland, submontane and montane forests and gallery forests. In Kenya, recorded from the W-C (Van Rompaey & Colyn, 2013).

**Genus** ***Civettictis*** Pocock, 1915. African Civet

306. ***Civettictis civetta*** (Schreber, 1776). English: African Civet. Swahili: Fungo. Chinese: 非洲灵猫. Recorded from sub-Saharan Africa from West Africa to the Horn of Africa down to southern Africa. In primary and secondary forests, woodlands and bushlands. In Kenya, widely distributed (Ray, 2013b).

**Family HYAENIDAE**

**Genus** ***Hyaena*** Brisson, 1762. Striped and Brown Hyaenas

307. ***Hyaena hyaena*** (Linnaeus, 1758). English: Striped Hyaena. Swahili: Fisi. Chinese: 鬣狗. Recorded from North and East Africa, including the Horn of Africa. In open habitats or light thorn bush country in arid to semi-arid environments. In Kenya, widespread in the N and S (Wagner, 2013).

**Genus** ***Crocuta*** Kaup, 1828. Spotted Hyaena

308. ***Crocuta crocuta*** (Erxleben, 1777). English: Spotted Hyaena. Swahili: Nyangao/Fisi. Chinese: 斑鬣狗. Recorded from many countries S of the Sahara. In a wide variety of habitats. In Kenya, widely distributed (East & Hofer, 2013).

**Genus** ***Proteles*** I. Geoffroy Saint-Hilaire, 1824. Aardwolf

309. ***Proteles cristatus*** (Sparrman, 1783). English: Aardwolf. Swahili: Fisi ya Nkole. Chinese: 土狼. Two subspecies with isolated population ranges; *Proteles c. cristatus* recorded from southern Africa and *Proteles c. Septentrionalis* in East Africa and the Horn of Africa. In open and grassland plains. In Kenya, recorded from the S, E and N (Anderson, 2013).

**Family HERPESTIDAE**

**Genus** ***Atilax*** F. G. Cuvier, 1826. Marsh Mongoose

310. ***Atilax paludinosus*** (G. Cuvier, 1829). English: Marsh Mongoose. Swahili: Nguchiro ya Maji. Chinese: 沼泽獴. Recorded from many countries in sub-Saharan Africa, except many parts of Namibia and Botswana. In riparian and estuarine habitats with suitable vegetation cover. In Kenya, recorded from W of the Rift Valley (Baker & Ray, 2013).

**Genus** ***Herpestes*** Illiger, 1811. Common Mongooses

311. ***Herpestes ichneumon*** (Linnaeus, 1758). English: Egyptian Mongoose. Swahili: Nguchiro. Chinese: 埃及獴. Recorded from sub-Saharan African countries, including some parts of North Africa. In lacustrine and riparian habitats, montane habitats and cultivated areas. In Kenya, widely distributed (Palomares, 2013).
312. ***Herpestes ochraceus*** (J.E. Gray, 1848). English: Somali Slender Mongoose. Swahili: Nguchiro. Chinese: 索马里貂獴. Recorded from the Horn of Africa. In semi-desert and desert habitats. In Kenya, recorded from the NE (Taylor, 2013).
313. ***Herpestes sanguineus*** (Rüppell, 1835). English: Slender Mongoose. Swahili: Nguchiro Mwembamba. Chinese: 草地貂獴. Recorded from many sub-Saharan African countries, except in some parts of Gabon and South Africa. In a wide variety of habitats, except in arid areas. In Kenya, widely distributed (Holfmann & Taylor, 2013).

**Genus** ***Bdeogale*** Peters, 1850. Bushy-tailed Mongooses

314. ***Bdeogale crassicauda*** Peters, 1852. English: Bushy-tailed Mongoose. Swahili: Nguchiro Kijivu/Kitu. Chinese: 毛尾臭獴. Recorded from a few countries in East and southern Africa. In *Acacia* and *Brachystegia* woodlands, montane and bamboo forests habitats. In Kenya, recorded in the E and SE (Taylor, 2013b).
315. ***Bdeogale jacksoni*** (Thomas, 1894). English: Jackson’s Mongoose. Swahili: Nguchiro. Chinese: 杰氏臭獴. Recorded from East Africa. In montane forests and bamboo zones. In Kenya, recorded from the C-W (Van Rompaey & Kingdon, 2013).
316. ***Bdeogale omnivora*** Heller, 1913. English: Sokoke Dog Mongoose. Swahili: Nguchiro ya Sokoke. Chinese: 索科凯臭獴. Recorded in Kenya and Tanzania. In coastal forests. In Kenya, recorded from N of Mombasa (Arabuko-Sokoke Forest) (Taylor, 2013c).

**Genus** ***Ichneumia*** I. Geoffroy Saint-Hilaire, 1837. White-tailed Mongoose

317. ***Ichneumia albicauda*** (G. Cuvier, 1829). English: White-tailed Mongoose. Swahili: Karambago. Chinese: 白尾獴. Recorded from many sub-Saharan African countries, including Egypt. In grasslands, savannas and wooded areas. In Kenya, widely distributed (Taylor, 2013d).

**Genus** ***Mungos*** E. Geoffroy Saint-Hilaire and F. G. Cuvier, 1795. Banded Mongooses

318. ***Mungos mungo*** (Gmelin, 1788). English: Banded Mongoose. Swahili: Nguchiro Miraba. Chinese: 缟獴. Recorded from many sub-Saharan African countries. In a wide range of habitats, especially savanna and woodlands, usually close to water. In Kenya, widely distributed (Cant & Gilchrist, 2013).

**Genus** ***Helogale*** Gray, 1861. Dwarf Mongooses

319. ***Helogale hirtula*** Thomas, 1904. English: Somali Dwarf Mongoose. Swahili: Kitafe. Chinese: 索马里矮獴. Recorded from E of the Rift Valley in Kenya, S Somalia, Ethiopia and South Sudan. In thickets and shrubby deciduous woodlands dominated by *Acacia* bushes (Kingdon & Van Rompaey, 2013).
320. ***Helogale parvula*** (Sundevall, 1847). English: Common Dwarf Mongoose. Swahili: Kitafe. Chinese: 纳塔尔矮. Recorded from southern Africa-East Africa, including the Horn of Africa. In open woodlands, thickets and wooded savannas with termitaria, rock outcrops, crevices or hollow logs for use as dens. In Kenya, widely distributed (Creel, 2013).

**ORDER PHOLIDOTA (Pangolins–three species)**

**Family MANIDAE**

**Genus** ***Phataginus*** Rafinesque, 1821. Tree Pangolins

321. ***Phataginus tricuspis*** (Rafinesque, 1821). English: Tree Pangolin. Swahili: Kakakuona ya Miti. Chinese: 树穿山甲. Recorded from West to East Africa, including Angola. In lowland tropical moist forests (primary and secondary), savanna/forest mosaics, cultivated and fallow fields. In Kenya, recorded from the W (Kingdon & Hoffmann, 2013c).

**Genus** ***Smutsia*** Gray, 1865. Ground Pangolins

322. ***Smutsia gigantea*** (Illiger, 1815). English: Giant Pangolin. Swahili: Kakakuona Mkubwa. Chinese: 大穿山甲. Recorded from West Africa, as well Central to East Africa. In lowland tropical moist and swamp forests and in forest-savanna-cultivation mosaics. In Kenya, recorded from the W (near the shores of Lake Victoria close to the Ugandan border) (Kingdon et al., 2013).
323. ***Smutsia temminckii*** (Smuts, 1832). English: Ground Pangolin. Swahili: Kakakuona. Chinese: 南非穿山甲. Recorded from southern to East Africa, including Sudan and Chad. In various woodland and savanna habitats, often with thick undergrowth, and in floodplain grasslands. In Kenya, recorded from W of the Rift Valley (Swart, 2013).

**ORDER PERISSODACTYLA (Odd-toed Ungulates–four species)**

**Family EQUIDAE**

**Genus** ***Equus*** Linnaeus, 1758. Zebras

324. ***Equus grevyi*** Oustalet, 1882. English: Grévy’s Zebra. Swahili: Punda Milia Somali. Chinese: 狭纹斑马. Recorded from the N (some Laikipia ranches) and S (Tsavo NPs (E and W) Kenya, and in Ethiopia. In arid and semi-arid grass/shrubland with permanent water (Williams S, 2013).
325. ***Equus quagga*** Boddaert, 1785. English: Plains Zebra. Swahili: Punda Milia. Chinese: 平原斑马. Recorded from southern to East Africa, including South Sudan and Ethiopia. In grasslands as well as grassland-bushland mosaics. In Kenya, widely distributed (Klingel, 2013a).

**Family RHINOCEROTIDAE**

**Genus** ***Ceratotherium*** Gray, 1867. White Rhinoceros

326. ***Ceratotherium simum*** (Burchell, 1817). English: White Rhinoceros. Swahili: Kifaru. Chinese: 白犀. Native to southern Africa but introduced to Uganda and Kenya. In savanna woodlands. In Kenya, introduced in parks, reserves and conservancies (e.g., Lake Nakuru and Ol Pejeta) (Owen-Smith, 2013a).

**Genus** ***Diceros*** Gray 1821. Black Rhinoceros

327. ***Diceros bicornis*** (Linnaeus, 1758). English: Black Rhinoceros. Swahili: Faru. Chinese: 黑犀. Recorded from Kenya, Tanzania and southern Africa. In a wide variety of habitats. In Kenya, found in selected protected areas (Nairobi NP, Lake Nakuru NP) and three private conservancies (Emslie & Adock, 2013).

**ORDER CETARTIODACTYLA (Even-toed Ungulates, Whales and Dolphins–63 species)**

**Family SUIDAE**

**Genus** ***Potamochoerus*** Thomas, 1904. Bushpig

328. ***Potamochoerus larvatus*** (F. Cuvier 1822). English: Bushpig. Swahili: Nguruwe Mwitu. Chinese: 假面野猪. Recorded from southern to East Africa, including DR Congo, South Sudan, Ethiopia and Somalia. In relatively dense vegetation types with available food, cover and water, in forests and riverine or xeric scrub forests and thicket formations (Seydack, 2013). In Kenya, widely distributed.

**Genus** ***Hylochoerus*** Thomas, 1904. Forest Hog

329. ***Hylochoerus meinertzhageni*** Thomas, 1904. English: Forest Hog. Swahili: Nguruwe Nyeusi/Senge: Chinese: 大林猪. Recorded from West to East Africa, including in Ethiopia. In a wide variety of forest types. In Kenya, recorded from C and W regions (highlands including Kakamega, Cherangani and Aberdares Ranges) (d’Huart & Kingdon, 2013).

**Genus** ***Phacochoerus*** F. Cuvier, 1826. Warthogs

330. ***Phacochoerus aethiopicus*** (Pallas, 1766). English: Desert Warthog. Swahili: Ngiri ya Somalia. Chinese: 疣猪. Recorded from E of the Rift Valley in Kenya, Somalia and Ethiopia. In open arid bushland and open woodland habitats (Grubb & d’Huart, 2013). In Kenya, recorded from the N (Turkana and Samburu) and E (Boni-Dodori Forest).
331. ***Phacochoerus africanus*** (Gmelin, 1788). English: Common Warthog. Swahili: Ngiri. Chinese: 非洲疣猪. Recorded from many African countries S of the Sahara, including Ethiopia, Djibouti and Eritrea. In savanna grassland, open bushland and woodland habitats. In Kenya, widely distributed (Cumming, 2013).

**Family HIPPOPOTAMIDAE**

**Genus** ***Hippopotamus*** Linnaeus, 1758. Common Hippopotamus

332. ***Hippopotamus amphibius*** Linnaeus, 1758. English: Common Hippopotamus. Swahili: Kiboko. Chinese: 河马. Recorded from many African countries. Mainly in major permanent rivers and lakes (Klingel, 2013b). In Kenya, widespread in permeant rivers (Mara, Tana, Athi, Ewaso Nyiro and Lake Naivasha).

**Family BALAENOPTERIDAE**

The Swahili name for whale is ‘Nyangumi’ and dolphin is ‘Pombo’. 

**Genus** ***Balaenoptera*** Lacépède, 1804. Rorquals (Baleen Whales)

333. ***Balaenoptera borealis*** Lesson, 1828. English: Sei Whale. Chinese: 鳁鲸. Worldwide distribution as well as in Kenya. In cold-temperate to warm-temperate waters (Reilly et al., 2008a).
334. ***Balaenoptera edeni*** Anderson, 1879. English: Bryde’s Whale. Chinese: 埃氏鳁鲸. Worldwide distribution in warm-temperate to tropical waters (Reilly et al., 2008b).
335. ***Balaenoptera musculus*** (Linnaeus, 1758). English: Blue Whale. Chinese: 蓝鲸. Worldwide distribution in arctic to tropical waters (Reilly et al., 2008c).
336. ***Balaenoptera physalus*** (Linnaeus, 1758). English: Fin Whale. Chinese: 长须鲸. Worldwide distribution in arctic to tropical waters (Reilly et al., 2013).

**Genus** ***Megaptera*** Gray, 1846. Humpback Whale

337. ***Megaptera novaeangliae*** (Borowski, 1781). English: Humpback Whale. Chinese: 座头鲸. Worldwide distribution in cold-temperate to tropical waters (Reilly et al., 2008d).

**Family DELPHINIDAE**

**Genus** ***Feresa*** Gray, 1870. Pygmy Killer Whales

338. ***Feresa attenuata*** (Gray, 1875). English: Pygmy Killer Whale. Chinese: 小逆戟鲸. Worldwide distribution in tropical to warm-temperate waters (Taylor et al., 2008a).

**Genus** ***Globicephala*** Lesson, 1828. Pilot Whales

339. ***Globicephala macrorhynchus*** Gray, 1846. English: Short-finned Pilot Whale. Chinese: 大吻巨头鲸. Worldwide distribution in tropical, warm-temperate waters and cold-temperate waters of the N Pacific (Taylor et al., 2011).

**Genus** ***Lagenodelphis*** Fraser, 1956. Fraser’s Dolphin

340. ***Lagenodelphis hosei*** Fraser, 1956. English: Fraser’s Dolphin. Chinese: 霍氏海豚. Worldwide distribution in warm-temperate to tropical waters (Hammond et al., 2012a).

**Genus** ***Orcinus*** Fitzinger, 1860. Killer Whale

341. ***Orcinus orca*** (Linnaeus, 1758). English: Killer Whale. Chinese: 逆戟鲸. Worldwide distribution in all seas and oceans (Reeves et al. 2017).

**Genus** ***Pseudorca*** Reinhardt, 1862. False Killer Whale

342. ***Pseudorca crassidens*** (Owen, 1846). English: False Killer Whale. Chinese: 伪虎鲸. Worldwide distribution in temperate to tropical waters (Taylor et al., 2008b).

**Genus** ***Sousa*** Gray, 1866. Humpback Dolphins

343. ***Sousa chinensis*** (Osbeck, 1765). English: Indo-Pacific Humpbacked Dolphin. Chinese: 中华白海豚. Restricted to Indian Ocean coastal waters and rivers from False Bay, South Africa, E to S China and Moreton Bay, Queensland (Jefferson et al., 2017).

**Genus** ***Stenella*** Gray, 1866. Oceanic Dolphins

344. ***Stenella attenuata*** (Gray, 1846). English: Pantropical Spotted Dolphin. Chinese: 弱原海豚. Worldwide distribution in temperate to tropical waters (Hammond et al., 2012b).
345. ***Stenella longirostris*** (Gray, 1828). English: Spinner Dolphin. Chinese: 长嘴海豚. Worldwide distribution in warm-temperate to tropical waters (Bearzi et al., 2012).

**Genus** ***Steno*** Gray, 1846. Rough-toothed Dolphin

346. ***Steno bredanensis*** (G. Cuvier in Lesson, 1828). English: Rough-toothed Dolphin. Chinese: 糙齿长吻海豚. Worldwide distribution in warm-temperate to tropical waters (Hammond et al., 2012c).

**Genus** ***Tursiops*** Gervais, 1855. Bottlenose Dolphins

347. ***Tursiops aduncus*** (Ehrenberg, 1833). English: Indo-Pacific Bottlenose Dolphin. Chinese: 印度洋宽吻海豚. Restricted to Indian Ocean (Hammond et al., 2012d).
348. ***Tursiops truncatus*** (Montagu, 1821). English: Common Bottlenose Dolphin. Chinese: 宽吻海豚. Worldwide distribution in temperate to tropical waters, including the Black Sea (Hammond et al., 2012e).

**Family PHYSETERIDAE**

**Genus** ***Kogia*** Gray, 1846. Sperm Whales

349. ***Kogia breviceps*** (Blainville, 1838). English: Pygmy Sperm Whale. Chinese: 小抹香鲸. Worldwide distribution in temperate to tropical waters (Taylor et al., 2012a).
350. ***Kogia sima*** (Owen, 1866). English: Dwarf Sperm Whale. Chinese: 矮抹香鲸. Worldwide distribution in warm-temperate to tropical waters occasionally strands in cold-temperate areas (Taylor et al., 2012b).

**Genus** ***Physeter*** Linnaeus, 1758. Toothed Sperm Whale

351. ***Physeter macrocephalus*** Linnaeus, 1758. English: Sperm Whale. Chinese: 抹香鲸. Worldwide distribution in antarctic and cold-temperate waters (northern hemisphere) to tropical waters (Taylor et al., 2008c).

**Family ZIPHIIDAE**

**Genus** ***Indopacetus*** Moore, 1968. Longman’s Beaked Whale

352. ***Indopacetus pacificus*** (Longman, 1926). English: Longman’s Beaked Whale. Chinese: 太平洋剑吻鲸. Indian Ocean and W South Pacific, including tropical waters (Taylor et al., 2008d).

**Genus** ***Mesoplodon*** Gervais, 1850. Beaked Whales

353. ***Mesoplodon densirostris*** (Blainville, 1817). English: Blainville’s Beaked Whale. Chinese: 瘤齿喙鲸. Worldwide distribution in temperate to tropical waters (Taylor et al., 2008e).
354. ***Mesoplodon ginkgodens*** Nishiwaki & Kamiya, 1958. English: Ginkgo-toothed Beaked Whale. Chinese: 杏齿喙鲸. North Pacific and Indian Oceans in warm-temperate to tropical waters (Taylor et al., 2008f).

**Family GIRAFFIDAE**

**Genus** ***Giraffa*** Brisson, 1762. Giraffe

355. ***Giraffa camelopardalis*** (Linnaeus, 1758). English: Giraffe. Swahili: Twiga. Chinese: 长颈鹿. Recorded from Central, East and southern Africa. In arid areas and savannas and *Acacia-Commiphora* woodlands. In Kenya, three sub-species recognized: *Giraffa c. rothschildi* Lydekker, 1903, Rothschild’s Giraffe, recorded from NW Kenya as far E and S as Lake Nakuru; *Giraffa c. reticulata* (Kingdon, 1997), Reticulated Giraffe, from E of the Rift Valley and N of Mt. Kenya and Tana River; and *Giraffa c. tipperlskirchi* (Kingdon, 1997), Maasai Giraffe from S Kenya (Ciofolo & Le Pendu, 2013).

**Family BOVIDAE**

**Genus** ***Syncerus*** Hodgson, 1847. African Buffalo

356. ***Syncerus caffer*** (Sparrman, 1789). English: African Buffalo. Swahili: Nyati/Mbogo. Chinese: 非洲水牛. Recorded from many countries in Africa S of the Sahara. In a wide variety of habitats. In Kenya, suspecies *Syncerus c. brachyceros* is widespread (Prins & Sinclair, 2013).

**Genus** ***Tragelaphus*** de Blainville, 1816. Spiral-horned Antelopes

357. ***Tragelaphus imberbis*** (Blyth, 1869). English: Lesser Kudu. Swahili: Tandala Mdogo. Chinese: 小林羚. Recorded from Tanzania, N and E Kenya, Ethiopia and S Somalia. In semi-arid areas with medium to dense woody cover, including riverine forests and thickets (Leuthold, 2013a). In Kenya, widely distributed.
358. ***Tragelaphus strepsiceros*** (Pallas, 1766). English: Greater Kudu. Swahili: Tandala/Tandala Mkubwa. Chinese: 扭角林羚. Recorded from southern to East Africa, including the Horn of Africa, Chad and Central African Republic. In a wide range of savanna vegetation types from dry thorn bush to mixed broad-leaved woodlands. In Kenya, recorded from the SE and N (Owen-Smith, 2013b).
359. ***Tragelaphus scriptus*** (Pallas, 1766). English: Bushbuck. Swahili: Mbawala/Pongo. Chinese: 薮羚. Recorded from many African countries S of the Sahara. In a wide variety of habitats. In Kenya, widely distributed (Plumptre & Wronski, 2013).
360. ***Tragelaphus spekii*** Speke, 1863. English: Sitatunga. Swahili: Nzohe. Chinese: 林羚. Recorded from central East and parts of southern Africa. In dense vegetation of perennial and seasonal swamps, marshy clearings within forests and riverine thickets. In Kenya, recorded from the W (Saiwa Swamp and papyrus swamps around Lake Victoria (May & Lindholm, 2013).
361. ***Tragelaphus euryceros*** (Ogilby, 1837). English: Bongo; Swahili: Ndongoro/Bongo; Chinese: 肯尼亚林羚. Two subspecies occur in African and only one in Kenya. *Tragelaphus e. isaaci* recorded from W and C Kenya (Mt. Kenya, Mau and Eburu forests, and Aberdare Ranges). In ecotone habitat, mainly in transitional vegetation at the forest edge in highland and montane areas (Elkan & Smith, 2013).
362. ***Tragelaphus oryx*** (Pallas, 1766). English: Common Eland. Swahili: Pofu. Chinese: 普通林羚. Recorded from southern to East Africa, including South Sudan and Ethiopia. In lowland and highland grasslands and savannas habitats. In Kenya, widely distributed (Thouless, 2013).

**Genus** ***Nesotragus*** C. H. Smith, 1827. Suni

363. ***Nesotragus moschatus*** Von Dueben, 1846. English: Suni. Swahili: Paa Mwekundu. Chinese: 岛羚. Recorded from E South Africa, Mozambique, SE Zimbabwe, S Malawi, Tanzania and Kenya. In thickets, forests and dense, evergreen woodlands. In Kenya, widely distributed (Hoffmann & Kingdon, 2013a).

**Genus** ***Philantomba*** Blyth, 1840. Blue Duikers

364. ***Philantomba monticola*** (Thunberg, 1789). English: Blue Duiker. Swahili: Ndimba/Chesi. Chinese: 蓝小羚羊. Recorded from Central, East and southern Africa. In forested and wooded habitats both in undisturbed and disturbed areas. In Kenya, recorded from the coastal strip and W (Hart & Kingdon, 2013).

**Genus** ***Sylvicapra*** Ogilby, 1836. Common Duiker

365. ***Sylvicapra grimmia*** (Linnaeus, 1758). English: Common Bush Duiker. Swahili: Nsya. Chinese: 灰小羚羊. Recorded from many African countries S of the Sahara. In savanna woodland habitats, especially in relatively open country and in the alpine zone in some mountainous areas. In Kenya, widely distributed (Wilson V, 2013).

**Genus** ***Cephalophus*** Hamilton Smith, 1827. Forest Duilers

366. ***Cephalophus adersi*** Thomas, 1918. English: Ader’s Duiker. Swahili: Mangi/Paa Nunga. Chinese: 艾氏小羚羊. Recorded from Kenya and Tanzania. In coastal forests, woodlands and thickets in undisturbed coastal habitats. In Kenya, recorded along the N coast in Arabuko-Sokoke Forest and Boni-Dodori Forest Reserves (Williams A, 2013).
367. ***Cephalophus harveyi*** (Thomas, 1893). English: Harvey’s Duiker. Swahili: Kiduku/Funo. Chinese: 哈氏小羚羊. Recorded from East Africa, including N Malawi, Zambia and Ethiopia. In moist coastal forests, riverine gallery forests and montane forests. In Kenya, recorded from coastal forests and C regions (Aberdare Ranges and Mt. Kenya) (Kingdon & Rovero, 2013).
368. ***Cephalophus nigrifrons*** Gray, 1871. English: Black-fronted Duiker. Swahili: Nsya. Chinese: 黑脸小羚羊. Recorded from Central Africa, as well as Uganda and Kenya. In lowland tropical rainforests, montane forests and moorland (Plumptre, 2013). In Kenya, recorded from C (Mt. Kenya and Aberdare Ranges) and W regions (Mt. Elgon).
369. ***Cephalophus weynsi*** Thomas, 1901. English: Weyns’s Duiker. Swahili: Funo. Chinese: 韦氏小羚羊. Recorded from Central to East Africa, including South Sudan. In undisturbed and disturbed highland forests. In Kenya, recorded from the W (Mt. Elgon, Kakamega and Mau Escarpment Forests) (Hart, 2013).
370. ***Cephalophus silvicultor*** (Afzelius, 1815). English: Yellow-backed Duiker. Swahili: Kipoke. Chinese: 黄背小羚羊. Recorded from West to Central Africa, as well as N Angola, Zambia and Kenya. In lowland and montane primary and secondary forests (Kingdon & Lahm, 2013p). In Kenya, recorded from the SW (Mau Forest, Mt. Elgon).

**Genus** ***Raphicerus*** C. H. Smith, 1827. Steenbok

371. ***Raphicerus campestris*** (Thunberg, 1811). English: Steenbok. Swahili: Isha. Chinese: 小岩羚. Two subspecies recorded in Africa, only one confirmed to occur in Kenya. *Raphicerus c. neumanni* from S Kenya and C Tanzania. In a wide variety of habitats (du Toit, 2013).

**Genus** ***Madoqua*** Ogilby, 1836. Dik-diks

372. ***Madoqua saltiana*** (Desmarest, 1816). English: Salt’s Dik-dik. Swahili: Digidigi. Chinese: 林犬羚. Recorded from the Horn of Africa, including Ethiopia and Kenya. In semi-desert scrub and deciduous bushlands. In Kenya, recorded from the N (Malkamari National Reserve) (Yalden, 2013c).
373. ***Madoqua kirkii*** (Günther, 1880). English: Kirk’s Dik-dik Species Group. Swahili: Digidigi/Suguya. Chinese: 柯氏犬羚. This species is treated as a species/subspecies complex, which provisionally includes four species (*M. kirkii, M. cavendishi*, *M. thomasi* and *M. damarensis*), with proposed subspecies within each of the four species (Brotherton, 2013). Molecular evidence strongly suggests that these are effectively full species, but the details of taxononmy and biogeography of the species remain uncertain, which is why members of this complex are placed under a single profile. The provisional distribution range of two species found in Kenya include; *Madoqua (k.) kirkii* mainly in SE-N Kenya and some parts in Tanzania; and *Madoqua (k.) cavendishi* mainly in S-NW Kenya and parts of Tanzania. In arid areas of scrub or open woodland (Brotherton, 2013).
374. ***Madoqua guentheri*** Thomas, 1894. English: Gunther’s Dik-dik. Swahili: Digidigi ya Pua Murefu. Chinese: 冈氏犬羚. Recorded from NE Uganda, Kenya, N to S Somalia and Ethiopia. In the driest, hottest and thickest thorn brush habitats (Hoppe & Brotherton, 2013). In Kenya, recorded from the N (from Lake Turkana, Laikipia to Mandera).

**Genus** ***Eudorcas*** Fitzinger, 1869. Ring-horned Gazelles

375. ***Eudorcas thomsonii*** (Günther, 1884). English: Thomson’s Gazelle. Swahili: Swala Tomi. Chinese: 托氏羚. Recorded from N Tanzania and C-S Kenya. In short grasslands and open wooded habitats (FitzGibbon & Wilmshurst, 2013). In Kenya, widely distributed.

**Genus** ***Nanger*** Lataste, 1885. Greater Gazelles

376. ***Nanger granti*** (Brooke, 1872). English: Grant’s Gazelle Species Group. Swahili: Swala Granti. Chinese: 格氏羚. In the past treated as a single species, now recognized as a species group consisting of three distinct species (*Nanger* (*g.*) *granti, Nanger* (*g.*) *notata* and *Nanger* (*g.*) *petersii*) based on the level of genetic differentiation (Siegismund et al., 2013), even though there is overwhelming evidence (Lorenzen et al., 2008) for raising the three groups to species level (Siegismund et al., 2013). The provisional distribution range of *Nanger* (*g.*) *granti* is mainly in E to N Kenya, and small ranges in Uganda, South Sudan, Ethiopia and Somalia; *Nanger* (*g.*) *notata* mainly in Tanzania and a small range in S Kenya; and *Nanger* (*g.*) *petersii* mainly in E Kenya and a small range in Somalia. Grant’s Gazelles occur in open savannas and *Acacia* and savanna woodlands (Siegismund et al., 2013).

**Genus** ***Litocranius*** Kohl, 1886. Gerenuk

377. ***Litocranius walleri*** (Brooke, 1879). English: Gerenuk. Swahili: Swala Twiga/Njoga. Chinese: 长颈羚. Recordesd from S Djibouti, East to S Ethiopia, much of Somalia and Kenya, including NE Tanzania. In bushland, thickets, semi-arid and arid thorn-bush habitats (Leuthold, 2013b). In Kenya, recorded from the E and N, especially in protected areas and private ranches.

**Genus** ***Ourebia*** Laurillard, 1842. Oribi

378. ***Ourebia ourebia*** (Zimmermnann, 1783). English: Oribi. Swahili: Taya. Chinese: 侏羚. Recorded from several countries in Africa S of the Sahara. In sloping grasslands and mixed woodlands maintained by fire and grazing. Two subspecies recognized in Kenya; *Ourebia o. cottoni* found in the SW and *Ourebia o. haggardi* (Haggard’s Oribi) in coastal forests N of Lamu (Boni-Dodori Forests); *Ourebia o. kenyae* occurred on the lower slopes of Mt. Kenya but is now extinct (Brashares & Arcese, 2013; Hillman et al., 1988).

**Genus** ***Redunca*** C. H. Smith, 1827. Reedbucks

379. ***Redunca fulvorufula*** (Afzelius, 1815). English: Mountain Reedbuck. Swahili: Tohe ya Milima. Chinese: 小苇羚. Three subspecies recognized in Africa but only one in Kenya. *Redunca f. chanleri* is recorded from W of the Rift Valley in Kenya, including Uganda, SE Sudan, Ethiopia and N Tanzania. In cool, mountainous regions above 1 500 m a.s.l. (Avenant, 2013).
380. ***Redunca redunca*** (Pallas, 1767). English: Bohor Reedbuck. Swahili: Tohe. Chinese: 塞内加尔小苇羚. Recorded from Senegal to Ethiopia, including East Africa. In woodlands and flood-plain grasslands. In Kenya, recorded in the SE-SW (Hoffmann & Kingdon, 2013b).

**Genus** ***Kobus*** Smith, 1840. Kobs

381. ***Kobus ellipsiprymnus*** (Ogilby, 1833). English: Waterbuck. Swahili: Kuro/Kobu/Kuru. Chinese: 水羚. This waterbuck is considered to have two subspecies (*Kobus e. ellipsiprymnus* and *Kobus e. defassa*) in Africa, both occurring in Kenya (Springe, 2013). *Kobus e. ellipsiprymnus* from Senegal to Ethiopia and some parts of southern Africa; *Kobus e. defassa* from southern to East Africa, including Somalia. In bushland and woodland habitats close to water (Springe, 2013). In Kenya, widely distributed, except for the N and NE.

**Genus** ***Oreotragus*** A. Smith, 1834. Klipspringer

382. ***Oreotragus oreotragus*** (Zimmermann, 1783). English: Klipspringer. Swahili: Mbuzi Mawe/Ngurunguru. Chinese: 山羚. Recorded from southern to East Africa, including Ethiopia, and South Sudan, Eritrea, N Somalia, Cameroon, Nigeria and Central African Republic. In a wide variety of habitats characterized by rocky stony ground and abundant short vegetation in rocky hillsides and escarpments. In Kenya, widely distributed (Roberts, 2013).

**Genus** ***Aepyceros*** Sundevall, 1845. Impala

383. ***Aepyceros melampus*** (Lichtenstein, 1812). English: Impala. Swahili: Swala Pala. Chinese: 黑斑羚. This impala is considered to have two subspecies in Africa, only one of which occurs in Kenya. *Aepyceros m. melampus* recorded from southern to East Africa. In light woodlands, savannas and open *Acacia* savanna habitats (Fritz & Bourgarel, 2013). In Kenya, widely distributed.

**Genus** ***Beatragus*** Heller, 1912. Hirola

384. ***Beatragus hunteri*** (Sclater, 1889). English: Hirola. Swahili: Hirola. Chinese: 亨氏大羚羊. Recorded from NE Kenya (Tana and Juba Rivers of E Kenya and N to Garissa; introduced population in E portions of Tsavo East NP) and W Somalia. In semi-arid thorn bush, open bush grassland, light woodland, lush savanna grassland and seasonally-flooded habitats (Butynski, 2013).

**Genus** ***Damaliscus*** Sclater and Thomas, 1894. Damalisks

385. ***Damaliscus lunatus*** (Burchell, 1824). English: Topi. Swahili: Nyamera. Chinese: 南非大羚羊. This topi is considered to have six subspecies recorded in different countries, with only three confirmed in Kenya. *Damaliscus l. jimela* (from East Africa in Great Lakes regions); *Damaliscus l. tiang* (SE Chad to SW Ethiopia and NW Kenya); and *Damaliscus l. topi* (in Kenya N of Malindi and S Somalia). In flood-plain habitats centered on wetlands (Duncan, 2013).

**Genus** ***Alcelaphus*** de Blainville, 1816. Hartebeests

386. ***Alcelaphus buselaphus*** (Pallas, 1766). English: Hartebeest. Swahili: Kongoni/Konzi. Chinese: 麋羚. There are eight subspecies considered for this hartebeest, with some having relatively large distribution ranges in Africa, with only three confirmed to occur in Kenya. *Alcelaphus b. cokii* (S Kenya and N Tanzania); *Alcelaphus b. lelwel* (SE Chad, Central African Republic, SE Ethiopia, N Kenya and NW Tanzania); and *Alcelaphus b. cokii* X* A. b. lelwel* (intergrade populations between these subspecies in Kenya) (Gosling & Capellini, 2013). In woodland-associated grasslands and savanna clearings.

**Genus** ***Connochaetes*** Lichtenstein, 1821. Wildebeest

387. ***Connochaetes taurinus*** (Burchell, 1823). English: Blue Wildebeest. Swahili: Nyumbu ya Montu. Chinese: 斑纹角马. There are five subspecies recognized under this species of wildebeest restricted to East and southern Africa. In relatively dry areas with short grass. Two subspecies occur in Kenya (Kingdon, 1997): *Connochaetes t. albojubatus* (recorded from the Athi Plains in S Kenya to the edge of Tsavo West and across N Tanzania to the N shores of Lake Tanganyika) and *Connochaetes t. mearnsi* confined to the Serengeti-Mara ecosystem and adjacent grasslands both in Kenya and Tanzania (Estes, 2013a).

**Genus** ***Hippotragus*** Sundevall, 1845. Roan and Sable Antelopes

388. ***Hippotragus equinus*** (É. Geoffroy, 1803). English: Roan Antelope. Swahili: Korongo. Chinese: 马羚. Recorded from West Africa to Ethiopian, and some parts in East and Central Africa. In savannas and woodlands. In Kenya, recorded from the W (Ruma NP) (Chardonnet & Crosmary, 2013).
389. ***Hippotragus niger*** (Harris, 1838). English: Sable Antelope. Swahili: Palahala/Mbarapi. Chinese: 貂羚. Recorded from southern and East Africa. In miombo (*Brachystegia*) woodlands. In Kenya, recorded from the SE (Shimba Hills NP and Forest Reserve) (Estes, 2013b).

**Genus** ***Oryx*** de Blainville, 1816. Oryxes

390. ***Oryx beisa*** (Rüppell, 1835). English: Beisa Oryx. Swahili: Choroa/Barabara. Chinese: 东非长角羚. Recorded from the Horn of Africa, from the Red Sea to Somalia, and East Africa, including South Sudan. In arid grasslands and bushlands. There are two subspecies recognized under this species that occur in Kenya: *Oryx b. beisa* (arid parts of Ethiopia and the Horn of Africa, SE Sudan and much of E and N Kenya); and *Oryx b. callotis* recorded from SE Kenya (S of the Tana River) and NE Tanzania (Wacher & Kingdon, 2013).

## DISCUSSION

Kenya has a rich diversity of mammalian species (390), representing about one third of the 1 116 mammal species recorded in Africa (Butynski et al., 2013; Happold D, 2013a; Happold M & Happold D, 2013; Kingdon & Hoffman, 2013a; Kingdon & Hoffman, 2013b; Kingdon et al., 2013). Comprehensive mammal surveys, inside and outside protected areas, are likely to yield additional species in the future. In addition, the taxonomy of some bats, rodents and shrews is still in a state of flux, and additional systematic work is likely to uncover species new to science and well as valid species currently treated as synonyms, thus adding to the list. Indeed, small mammals and primates comprise taxonomic groups where large numbers of new species have been discovered in recent years (Reeder et al., 2007). In conclusion, this is the first attempt to compile a comprehensive list of the mammals of Kenya. It is likely that the number of species will be revised in the future with increasing surveys and taxonomic revisions, particularly among small mammals.
